# Identification and Forecasting in Mortality Models

**DOI:** 10.1155/2014/347043

**Published:** 2014-06-02

**Authors:** Bent Nielsen, Jens P. Nielsen

**Affiliations:** ^1^Department of Economics, University of Oxford, Oxford OX1 2JD, UK; ^2^Programme on Economic Modelling, INET, University of Oxford, Oxford OX1 2JD, UK; ^3^Nuffield College, Oxford OX1 1NF, UK; ^4^Cass Business School, City University London, 106 Bunhill Row, London EC1Y 8TZ, UK

## Abstract

Mortality models often have inbuilt identification issues challenging the statistician. The statistician can choose to work with well-defined freely varying parameters, derived as maximal invariants in this paper, or with ad hoc identified parameters which at first glance seem more intuitive, but which can introduce a number of unnecessary challenges. In this paper we describe the methodological advantages from using the maximal invariant parameterisation and we go through the extra methodological challenges a statistician has to deal with when insisting on working with ad hoc identifications. These challenges are broadly similar in frequentist and in Bayesian setups. We also go through a number of examples from the literature where ad hoc identifications have been preferred in the statistical analyses.

## 1. Introduction

Mortality models are commonly used in a wide range of fields such as actuarial sciences, epidemiology, and sociology. They are often used in important decisions such as how to deal with unisex legislation in the pension industry; see Ornelas et al. [1] and Jarner and Kryger [2]. However, such models do often have inbuilt identification issues stemming from overparametrisation. While identification issues are omnipresent in statistical modelling, this paper focuses on mortality modelling, where estimated parameters are treated as time series and extrapolated to give forecasts of future mortality. The underlying theme of this paper is to provide strategies of avoiding arbitrariness resulting from the identification process. We suggest two ways forward. First, we can reparametrise the model in terms of a freely varying parameter, which therefore has to be of lower dimension than the original parameter. Secondly, we can work with an identified version of the original parameter as long as we keep track of the consequences of the identification choice. That way we ensure that two researchers making different identification choices get the same statistical inferences and forecasts.

A simple example is the age-period model for an age-period array of mortality rates. It is well-known that the levels of the age- and period-effects cannot be determined from the likelihood representing the overparametrisation of the model. When the estimated age- and period-effects are treated as time series and subjected to plotting and extrapolation, then our approach ensures that the statistical analysis is the same for two researchers identifying the above model in two different ways. Whereas this issue is relatively simple for the age-period model, identification becomes more tricky for complicated models such as the age-period-cohort model and the model of Lee and Carter [3], let alone two-sample situations.

Mortality models are built as a combination of age, period, and cohort-effects, but the likelihood only varies with a surjective function of these time effects. The time effects can be divided into two parts. One part that moves the likelihood function and another part which does not induce variation in the likelihood function. We will argue that all inferences and forecasts should be concerned primarily with the part of the parameter that moves the likelihood function. This does not preclude the researcher from working with the time effects, but it gives some limitations on what can be done. This is important because the motivation and the intuition of mortality models typically originate in the time effects. For instance, in the context of an age-period-cohort model linear trends cannot be identified so time series plots of the time effects need to be invariant to linear trends and extrapolations of time effects must preserve the arbitrary linear trend in the time effects. This applies regardless of whether the identification issue is dealt with in a frequentist manner or by Bayesian methods.

To formalise the discussion slightly return to the age-period example. Denote the predictor for the age-period data array by *μ*. The age-period model then determines how the predictor *μ* varies with a vector *θ* summarising age and period effects. That vector is split into two components *ξ* and *λ* so that the predictor only depends on *θ* through *ξ* but not on *λ* which cannot be identified by statistical analysis. In the age-period example *ξ* could reflect the contrasts and the overall level of the predictor *μ*, whereas *λ* reflects the level of the age effect. The more principled solution is then to work exclusively with *ξ* and simply consider *θ* as a motivation rather than the objective of the analysis. Another solution is to ad hoc identify *λ* based on a notion of mathematical convenience or based on a particular purpose given the substantive context.

Once an ad hoc identification of *λ* is chosen the identification problem appears to go away, because the likelihood analysis can now go through. The reason is that the variation of *θ* is now reduced to the variation of *ξ* precisely because *λ* is fixed. Suppose two researchers choose the same likelihood and the same parametrisation of *ξ* but different ad hoc identifications *λ* ^†^ and *λ* ^‡^. Which of their conclusions will be the same and which will be different? As the likelihood only depends on *ξ* the fits of the two researchers will be identical. But differences might arise if the statistical inference or forecasting or any other statistical analysis involves *λ* in some way.

Indeed, with many extrapolation methods forecasts will be invariant to the choice of *λ*. But, there will also be extrapolation methods where this is not the case. Examples arise in the age-period-cohort model, where linear trends have to be handled with care.

We will start by analysing linearly parametrised models at a rather general level. We do this with two aspects in mind. First, we need to step back to a point in the analysis before ad hoc identification is made. Secondly, we also want to avoid the discussion of how to choose *ξ* and *λ*, which tend to be specific to the mortality model in question. Working at the general level we can focus on the mappings between different parametrisations and the invariance properties coming from these mappings. It is then seen that the parameter *ξ* arises as a maximal invariant. The general setting also allows the formulation of a series of results discussing different types of ad hoc identification, first in a frequentist fashion and then in a Bayesian fashion.

Subsequently, we will consider the age-period-cohort model in detail, both for one- and two-sample situations. Using the general results it becomes easier to see that a number of popular methods inadvertently include features that are not invariant to ad hoc identification. These include the “intrinsic estimator” advocated by Yang et al. [4], the “mixed model approach” by Yang and Land [5], the Bayesian approach by Berzuini and Clayton [6], and the two-sample analysis by Riebler and Held [7]. Finally, we consider the nonlinearly parametrised model of Lee and Carter [3]. The nonlinearity gives a further complication since the mapping from the time effects to the mortality predictor is nondifferentiable. As it turns out the mortality predictor varies in a smooth space, so the nondifferentiability is avoided by working directly with the mortality predictor instead of the original time effects. Instead, a Lee-Carter application should consider whether a certain matrix has rank of unity or zero. Apart from that the analysis is similar to that of linearly parametrised models. Likewise a theory is given for two-sample situations.

Throughout the paper our concern rests exclusively with the identification problem and the consequences of ad hoc identification for estimation, plots, inference, and forecasting. In practice, important additional concerns are how to choose appropriate models and forecasting methods. We would like to refer to Girosi and King [8], Pitacco et al. [9] for general discussions of these issues, and also to Kuang et al. [10] and Coelho and Nunes [11] for discussions of forecast methods in the light of structural breaks. Instead, the aim of the paper is to present an overall framework that can help streamlining the identification discussion that has appeared in so many papers in so many fields over so many years.

Section 2 of this paper considers standard linear statistical models, which lend themselves to a relative straightforward analysis based on linear algebra. Any ad hoc identification splits the time effect into two components. The first component is an arbitrary component, which is not needed for the identification of the likelihood. The other component is necessary and sufficient to identify the model and hence sufficient for statistical analysis. In Section 3 it is outlined how to analyze the statistical model when the latter component is ad hoc identified. It is argued that this can cause difficulties for estimation, interpretation, and forecast. In Section 4 it is shown that Bayesian analysis shares the same challenges as the frequentist approach. In Sections 5 and 6 we study the two particular examples: the omnipresent age-period-cohort and Lee-Carter mortality models. All proofs are collected in the Appendix.

## 2. Statistical Models with Linear Parametrisations

In this section we present the identification problem in a linear framework. The problem is solved by analysing the mapping from the original time effect to the predictor which, in turn, leads to standard statistical analysis. In Section 6 we show how these ideas transfer to a nonlinear context. This contrasts with Section 3 in which we illustrate the analytical challenges and inconveniences arising from ad hoc identification.

In Section 2.1 we present the overparametrized linear model for the mortality predictor. The identification problem is defined in Section 2.2 via the likelihood. In an overparametrized linear model two different parameters might produce the same likelihood. In Section 2.3 we analyze the mapping from the overparametrised parameter to the predictor. This mapping enables us to split the overparametrised parameter into two. One arbitrary parameter and one parameter identify the model without being overparametrised. This latter parameter is shown to be a maximal invariant parameter. In Section 2.4 it is demonstrated how any statistical analysis can be based on this maximal invariant parameter alone. In particular we comment that visual data representations, hypothesis testing, and forecasting are simple and well defined. This in turn leads to standard statistical analysis.

The analysis of the linearly parametrised involves projections on linear or affine spaces and on their orthogonal complements. It is therefore convenient to introduce the following notation. A matrix *m* has full column rank if *m*′*m* is invertible. In this case the orthogonal complement *m*_⊥_ is a matrix so *m*_⊥_′*m* = 0 and (*m*, *m*_⊥_) is invertible. Thus, when *m* itself is invertible then *m*_⊥_ is the empty matrix. It is not difficult to calculate *m*_⊥_ in practise, an explicit construction of *m*_⊥_ follows from a singular value decomposition of *mm*′, choosing *m*_⊥_ as the eigenvectors associated with the zero eigenvalues. Moreover, let $\bar{m}=m{\left(m^{\prime }m\right)}^{-1}$ so that ${\bar{m}}^{\prime }m$ is the identity matrix, while ${\bar{m}}_{⊥}=m_{⊥}{\left(m_{⊥}^{\prime }m_{⊥}\right)}^{-1}$.

### 2.1. The Model

Think of the time effect *θ* as our preferred intuitive, but unidentified parameter, and think of the predictor *μ* as some function of *θ* specifying the model at hand. In a Poisson type model, where the mean specifies the distribution, *μ* could be the log of that mean. Such Poisson models are omnipresent in mortality models. We will often think of *θ* as containing some time effects. Often forecasting is carried out simply by isolating and extrapolating such a time effect.

Consider a data vector *Y* of dimension *n*. This could, for instance, be the vector consisting of the stacked mortality rates for a rectangular age-period array of dimension *I* × *J* in which case *n* = *IJ*. The statistical model for *Y* could be a generalized linear model. This involves an appropriately chosen distribution and a link function, which links the expected mortality rate to an *n*-dimensional predictor, which is denoted by *μ*. Taken together this defines a likelihood function *L*(*μ*; *Y*).

The model for the predictor *μ* is constructed in terms of, for instance, age, period, and cohort time effects. These time effects are summarized in a vector *θ*, which is of dimension *q* < *n*. Therefore *μ* is a surjective function of *θ*. For the moment the specification of the predictor is assumed linear so that

(1) $$\begin{array}{c} \;\mu =D\theta \;\text{for}\;\;\theta \in \Theta =R^{q}, \end{array}$$

for some design matrix *D* ∈ *R* ^*n*×*q*^. We refer to this specification as the mortality model, while the space Θ is the time effect space. The time effect space is chosen as an unrestricted real space in accordance with the starting point of most mortality analyses.

The parameter space for the likelihood function and therefore for the statistical model is given by the range of variation for the predictor *μ*; that is,

(2) $$\begin{array}{c} M=\left(\mu \in R^{n}:\mu =D\theta \;\;\text{for}\;\;\theta \in \Theta =R^{q}\right). \end{array}$$

The likelihood function is assumed uniquely identified on this space in the sense that for all pairs of predictors so *μ* ^†^ ≠ *μ* ^‡^ then the likelihood of *μ* ^†^, *μ* ^‡^ differ; that is,

(3) $$\begin{array}{c} L\left(\mu^{†};Y\right)\neq L\left(\mu^{‡};Y\right), \end{array}$$

for *Y* in a set with positive probability.

### 2.2. The Identification Problem

The identification problem of mortality models arises when the mapping from the time effect space Θ to the parameter space *M* is surjective but not injective. With a linear parametrisation this arises when the design matrix *D* has reduced column rank *p* < *q* so *D*′*D* is singular. In this situation there exists time effects *θ* ^†^ ≠ *θ* ^‡^ with the same likelihood:

(4) $$\begin{array}{c} L\left(D\theta^{†};Y\right)=L\left(D\theta^{‡};Y\right), \end{array}$$

for all data *Y*. Then the time effect space Θ is not useful as parameter space for the statistical model.

### 2.3. Analysing the Mapping *θ* ↦*μ*

When analysing the mapping from our intuitively preferred parametrisation *θ* into the linear predictor *μ*, we will be able to rewrite *θ* as a sum of two components: one is a function of the predictor and the other is the arbitrary part varying with *θ*, but not with the predictor. We provide two methods for analysis.

The first method is to find a basis *X* ∈ *R* ^*n*×*p*^ with full column rank⁡*p* for the design *D*. The design matrix of the mortality model can then be expressed as *D* = *XA*′ for some matrix *A* ∈ *R* ^*q*×*p*^ with full column rank⁡*p*. Introduce a new *p*-dimensional parameter:

(5) $$\begin{array}{c} \xi =A^{\prime }\theta . \end{array}$$

The parameter space *M* can then be written more parsimoniously as

(6) $$\begin{array}{c} M=\left(\mu \in R^{n}:\mu =X\xi \;\;\text{for}\;\;\xi \in R^{p}\right). \end{array}$$

The mapping from *ξ* to *μ* is bijective, so the statistical model can just as well be parametrised in terms of *ξ* ∈ *Ξ* = *R* ^*p*^.

Alternatively, the identification problem can be expressed through an invariance argument. This argument relates to the parameterization but resembles the classical invariance argument for reduction of data; see Cox and Hinkley [12, page 157]. With a linear parametrisation the argument involves the orthogonal complement to the matrix *A*. That is a matrix *A*_⊥_ ∈ *R* ^*q*×(*q*−*p*)^ which has the properties that *A*_⊥_′*A* = 0 and that (*A*, *A*_⊥_) is invertible. The mortality model (1) is defined by the mapping

(7) $$\begin{array}{c} \theta ⟼\mu =D\theta =XA^{\prime }\theta , \end{array}$$

from Θ = *R* ^*q*^ to *M*. This mapping is surjective in that two different values of *θ* may result in the same *μ* and therefore the same likelihood. These equivalence classes in the time effect space can be described by the group of transformations

(8) $$\begin{array}{c} g:\theta ⟼\theta +A_{⊥}\zeta , \end{array}$$

acting on Θ for arbitrary *ζ* ∈ *R* ^*q*−*p*^. Indeed, it holds that *θ* and *g*(*θ*) will result in the same *μ*. The mapping (7) is therefore invariant to the group *g*. We will argue that the parameter *ξ* = *A*′*θ* is a maximal invariant to the group *g* acting on Θ, which provides a link with (6). It has to be argued that for any *θ* ^†^, *θ* ^‡^ so that *ξ* ^†^ = *A*′*θ* ^†^ equals *ξ* ^‡^ = *A*′*θ* ^‡^ then *θ* ^‡^ = *g*(*θ* ^†^), see Cox and Hinkley [12, page 159]. For this argument use the orthogonal projection identity to write

(9) $$\begin{array}{c} \theta =A{\left(A^{\prime }A\right)}^{-1}\xi +A_{⊥}{\left(A_{⊥}^{\prime }A_{⊥}\right)}^{-1}\varphi ; \end{array}$$

for unique *ξ* = *A*′*θ* and *φ* = *A*_⊥_′*θ*. Thus, if *A*′*θ* ^‡^ = *A*′*θ* ^†^ then *θ* ^‡^ = *g*(*θ* ^†^) with *ζ* = *φ* ^‡^ − *φ* ^†^ = *A*_⊥_′(*θ* ^‡^ − *θ* ^†^).

In applications it can be difficult to find a basis *X* for the design *D*. It can be easier to find a group *g* and hence *A*_⊥_ and then use this information to construct *A* and a candidate basis $X=D\bar{A}$, noting that *D* = *XA*′. This argument leaves it to be proven that *X* is a basis, or equivalently, that the suggested group *g* actually describes the equivalence classes of the mapping from *θ* to *μ*.

It is useful to note that in the choices of *X*, *A* only the spaces spanned by them are unique since *XA*′ = *X* *mm* ^−1^ *A*′ for any invertible *m* ∈ *R* ^*p*×*p*^. Likewise, the maximal invariant *ξ* is only unique up to bijective transformations. This lack of uniqueness has no impact on the analysis of the likelihood albeit it influences interpretations.

### 2.4. Statistical Analysis Using the Maximal Invariant Parameter

The statistical model parametrised with the maximal invariant parameter *ξ* can be analysed by standard statistical techniques. This contrasts to a range of problems that arise when working with an ad hoc identified time effect *θ*. In the following the relatively simple standard statistical analysis of the model parametrised by *ξ* is discussed with respect to likelihood theory, interpretation, plots, hypothesis testing, forecasting, and Bayesian analysis. In Sections 3 and 4 we give an overview of the much more complicated theory underpinning models parametrised by the ad hoc identified time effect *θ*. Age-period-cohort examples follow in Section 5.

#### 2.4.1. Exponential Family Theory

Suppose the likelihood is drawn from a generalized linear model based on an exponential family. Then the model is actually a regular exponential family where the maximal invariant parameter *ξ* is the canonical parameter since it is freely varying in a real space; see Barndorff-Nielsen [13, page 116]. This opens up for a wealth of convenient statistical properties such as a likelihood equation with a simple expression and explicit conditions for a unique solution. In contrast, ad hoc identified parameters are based on an injective mapping of the canonical parameter *ξ* into *θ*; see Sections 3.1 and 3.2. It is then more difficult to fully exploit the exponential family theory.

#### 2.4.2. Interpretation and Plots

The maximal invariant parameter *ξ* varies freely in *R* ^*p*^. It can therefore be interpreted as the parameter of any standard statistical model. Since *ξ* is freely varying the coordinates of *ξ* can be interpreted independently. When *θ* is a collection of time effects then *ξ* can be organised as a collection of time series. Since the coordinates of *ξ* are freely varying the time series plots of the components of *ξ* have the usual interpretation of time series. In contrast, ad hoc identified estimators are constrained to a *p*-dimensional subspace Θ_*λ*_ of Θ = *R* ^*q*^, which is often affine but can be more complicated. A consequence is that plots are complicated to evaluate; see Section 3.4.1.

#### 2.4.3. Hypothesis Testing

Hypotheses are easily formulated and analysed when using the maximal invariant parametrisation. An affine hypothesis that restricts *ξ* to vary in a *p*_*H*_-dimensional affine subspace can be formulated as *H*′*ξ* = *η* for known matrices *H* ∈ *R* ^*p*×(*p*−*p*_*H*_)^, *η* ∈ *R* ^*p*−*p*_*H*_^. This implies a restriction on the predictor *μ* = *Xξ* of (6). Form the orthogonal complement *H*_⊥_ and recall the orthogonal projection identity $I_{n}=\bar{H}H^{\prime }+H_{⊥}{\bar{H}}_{⊥}^{\prime }$ so that $\mu =X\bar{H}H^{\prime }\xi +XH_{⊥}{\bar{H}}_{⊥}^{\prime }\xi$. Introduce a *p*_*H*_-dimensional parameter $\varphi ={\bar{H}}_{⊥}^{\prime }\xi$, a design matrix *X*_*H*_ = *XH*_⊥_, and an offset $Z_{H}=X\bar{H}\eta$. The restricted parameter space is

(10) $$\begin{array}{c} M_{H}=\left(\mu \in R^{n}:\mu =X_{H}\varphi +Z_{H}\;\;\text{for}\;\;\varphi \in R^{p_{H}}\right). \end{array}$$

In an exponential family context both the unrestricted model and the restricted model form regular exponential families. A variety of nice properties then follow for the estimators and the test statistics from the exponential family theory. Examples are given in Sections 5.3 and 5.5.3. In contrast, the hypothesis derived from restrictions on ad hoc identified parameters and the resulting degrees of freedom are complicated to analyse; see Section 3.4.2.

#### 2.4.4. Forecasting

Most often the objective of a mortality study is to forecast the future mortality. In the linear context, *μ* = *Xξ*, this is done by extending the design *X* and by extrapolating *ξ*.

It is usually easy to extend the design *X* into the forecast horizon. This involves the construction of a triangular block matrix with an appropriate number of extra rows corresponding to the data over the forecast horizon as well as extra columns representing the extra parameters that would be needed:

(11) $$\begin{array}{c} X^{h}=\left(\begin{array}{cc} X & 0\\ X_{1}^{h} & X_{2}^{h} \end{array}\right). \end{array}$$

Extrapolating *ξ* into a vector $\tilde{\xi }$ then gives the forecast

(12) $$\begin{array}{c} \tilde{\mu }=\left(X_{1}^{h},X_{2}^{h}\right)\left(\begin{array}{c} \xi \\ \tilde{\xi } \end{array}\right). \end{array}$$

The extrapolation of the parameter *ξ* can be done as follows. The estimated parameter, or part of it, can be thought of as a time series. Any forecast techniques from the time series literature applied directly to *ξ* can be used, subject to the usual contextual considerations.

Ad hoc identified time effects can be extrapolated in a similar way; see Section 3.4.3. This may, however, result in avoidable arbitrary effects in the forecast. Necessary and sufficient conditions for this eventuality are given for age-period-cohort models in Section 5.4.3. The practical examples are mainly Bayesian in nature and are discussed next.

#### 2.4.5. Bayesian Analysis

The introduction of the canonical parameter shows that the likelihood, in Bayesian notation, is of the form *p*(*y* | *θ*) = *p*(*y* | *ξ*) where *ξ* is freely varying. A purist Bayesian analysis can simply introduce a prior on the canonical parameter, *p*(*ξ*). This is updated in a straight forward way, resulting in the posterior *p*(*ξ* | *y*) = *p*(*y* | *ξ*)*p*(*ξ*)/*p*(*y*).

In contrast, introducing a prior on ad hoc identified parameters gives various difficulties. Only parts of the prior are updated by the likelihood, so that it becomes unclear which information arises from the data and which information arises from the ad hoc identification. Moreover, avoidable arbitrariness is introduced in the forecast; see Section 4. Introduction of hyperparameters exacerbates the issue. Examples are given in Sections 5.4.4, 5.5.2, and 6.1.6.

## 3. Working with the Time Effects

In Section 2 we considered the situations where estimation, hypothesis testing a hypothesis, or forecasting is carried out using the canonical parameter. However, there might be situations, where the original time effect parametrisation is preferred, perhaps because it is felt that this parametrisation is particularly helpful in guiding the intuition. This requires ad hoc identification of the time effect. In this section we will guide the considerations a statistician that has to go through when insisting on an analysis based on some nonunique parametrisations. As in Section 2 we focus on linearly parametrised models. Specific examples follow in Sections 5 and 6.

In Section 3.1 ad hoc identification is defined. As an example we consider a least squares estimation problem with collinear regressors in Section 3.2. For the age-period-cohort model reviewed in Section 5 it is common to ad hoc identify in two steps: first identifying levels then the linear trends. We consider such two-step ad hoc identification in Section 3.3. The consequence of ad hoc identification is considered in Section 3.4. Indeed, when forecasting the time effect, we do not want the forecast to depend on the identification scheme. The same applies to graphical visualisation of our data, where the eye may extract patterns that depend on the identification scheme. Likewise, confusion may arise when formulating a hypothesis directly on the time effect parameters.

### 3.1. Ad Hoc Identification

In this section the time effect parametrisation is considered. An identification scheme has to be introduced when working with the time effects. This may rest on mathematical convenience or it may be chosen for a particular purpose given the substantive context. We therefore call it ad hoc identification. Here we consider a simple identification scheme but turn to a more common two-step identification scheme in Section 3.3.

Once the canonical parameter *ξ* has been estimated there is often a wish to return to the original time effect *θ*. The two are linked through the surjective mapping

(13) $$\begin{array}{c} \theta ⟼\xi =A^{\prime }\theta , \end{array}$$

from Θ = *R* ^*q*^ to *Ξ* = *R* ^*p*^. Indeed, since *ξ* is constructed as a function of *θ* the notation for *ξ* is often chosen to reflect *θ*. The canonical parameter *ξ* does, however, only give partial information about *θ*. The remaining part, say *λ*, of *θ* will have to be chosen by the researcher and combined with *ξ*.

A linear ad hoc identification of *θ* comes about by the researcher choosing a constraint

(14) $$\begin{array}{c} L^{\prime }\theta =\lambda \end{array}$$

for some known *λ* ∈ *R* ^*q*−*p*^ and some matrix *L* ∈ *R* ^*q*×(*q*−*p*)^ chosen so the square matrix (*A*, *L*) is invertible. The time effect space Θ is now reduced to an affine subspace

(15) $$\begin{array}{c} \Theta_{\lambda }=\left(\theta_{\lambda }\in \Theta :L^{\prime }\theta_{\lambda }=\lambda \right). \end{array}$$

Given *θ* we can find *ξ*, *λ* through (13) and (14) as (*ξ*′,*λ*′)′ = (*A*,*L*)′*θ*. At the same time, given values of *ξ*, *λ* and the invertibility of (*A*, *L*), the ad hoc identified time effect is found through

(16) $$\begin{array}{c} \theta_{\lambda }={\left(\begin{array}{c} A^{\prime }\\ L^{\prime } \end{array}\right)}^{-1}\left(\begin{array}{c} \xi \\ \lambda \end{array}\right)=L_{⊥}{\left(A^{\prime }L_{⊥}\right)}^{-1}\xi +A_{⊥}{\left(L^{\prime }A_{⊥}\right)}^{-1}\lambda . \end{array}$$

In this notation a subindex *λ* is introduced to avoid confusion with the time effect *θ* in the original mortality model. Indeed, there are now four different parameters in play, namely, the original time effect *θ* ∈ Θ, the predictor *μ* ∈ *M*, the maximal invariant parameter *ξ* ∈ *Ξ* and the ad hoc identified time effect *θ*_*λ*_ ∈ Θ_*λ*_, each of which has a different interpretation. The mapping from *θ* to each of *μ*, *ξ*, and *θ*_*λ*_ is surjective, while there are bijective mappings between the latter three. The interpretations of the time effect *θ* and the canonical parameter *ξ* will inevitably be different. For a start they have different dimensions. Endowing the spaces with Euclidean norms shows that distances in the two spaces Θ and *Ξ* will be judged differently. The time effect *θ* and the ad hoc identified time effect *θ*_*λ*_ will similarly have different interpretations. Although they have the same dimensions the Euclidean norms on Θ and Θ_*λ*_ will be rather different. Confusion may arise in the interpretation of a mortality analysis if there is no clear distinction between *θ* and *θ*_*λ*_. In addition an unnecessary arbitrariness may arise when making inference on *θ*_*λ*_ or extrapolating *θ* ∈ Θ_*λ*_. We will return to these issues in Section 3.4.

It is perhaps interesting to note that despite the linear parametrisation the ad hoc identification need not be done in a linear fashion as in (14). Indeed it is common for Poisson models with a log link to ad hoc identify *θ* through the original multiplicative scale. That means that the ad hoc identification is done nonlinearly through

(17) $$\begin{array}{c} \xi =A^{\prime }\theta_{\lambda },\;\lambda =f\left(\theta_{\lambda }\right). \end{array}$$

The fit of the model is unaffected by the ad hoc identification. Indeed the fit is measured in terms of the estimate of the predictor *μ* = *Dθ*_*λ*_ where *D* = *XA*′. Since the identification is made so *ξ* = *A*′*θ*_*λ*_; the estimated predictor reduces to

(18) $$\begin{array}{c} \hat{\mu }=D{\hat{\theta }}_{\lambda }=XA^{\prime }{\hat{\theta }}_{\lambda }=X\hat{\xi }, \end{array}$$

regardless of the choice of ad hoc identification.

### 3.2. A Least Squares Example

As an illustration of estimation in the presence of ad hoc identification consider a normal likelihood. Different, but equivalent, expressions can be found depending on the parametrisation. The likelihood of the predictor *μ* is

(19) L(μ,σ2;Y)=(2πσ2)−n/2exp⁡{−12σ2(Y−μ)′(Y−μ)}for  μ∈M, σ2>0.

Rewriting it in terms of the canonical parameter it is

(20) L(ξ,σ2;Y)=(2πσ2)−n/2exp⁡⁡{−12σ2(Y−Xξ)′(Y−Xξ)}for  ξ∈Ξ=Rp, σ2>0,

while introducing the time effect parameter gives

(21) L(θ,σ2;Y) =(2πσ2)−n/2exp⁡{−12σ2(Y−XA′θ)′(Y−XA′θ)}for  θ∈Θ=Rq, σ2>0.

The likelihood (20) of the canonical parameter *ξ* can be analysed by the least squares method since the design *X* has full column rank. The maximum likelihood estimator for *ξ* and the predictor for the data are

(22) $$\begin{array}{c} \hat{\xi }={\left(X^{\prime }X\right)}^{-1}X^{\prime }Y,\;\;\hat{Y}=X\hat{\xi }=X{\left(X^{\prime }X\right)}^{-1}X^{\prime }Y. \end{array}$$

Along with the residual variance this is all the information that is given by the likelihood.

The likelihood (21) of the time effect *θ* only depends on *θ* through *ξ* = *A*′*θ*. The lack of identification means that the maximum likelihood estimator for *θ* has an arbitrary element, so that it is a set valued estimator. Based on (16) this can be expressed by

(23) Θ^λ=L⊥(A′L⊥)−1ξ^+A⊥(L′A⊥)−1λwhere  θ^λ∈Θλ⊂Θ,

for any *L* so (*A*, *L*) is invertible and for any *λ* ∈ *R* ^*q*−*p*^. The fit, however, remains the same and (18) becomes

(24) μ^=Dθ^λ=XA′{L⊥(A′L⊥)−1ξ^+A⊥(L′A⊥)−1λ}=Xξ^=Y^.

In order to compute actual estimates then *L*, *λ* have to be chosen, which amounts to ad hoc identification. For instance, with the ad hoc identifying restrictions *L* = *A*_⊥_ and *λ* = 0 then ${\hat{\theta }}_{\lambda }$ can be thought of as the least squares estimator of *Y* on *D* using the Moore-Penrose generalised inverse for the singular matrix *D*′*D*; see Searle [14, page 212]. See Section 5.4.1 for an example.

### 3.3. Step-Wise Identification

It is common to ad hoc identify parameter in a step-wise fashion. In the first step the time effect parameter is only partially constrained. The full identification then follows in a second step. An example is given in Section 5.4.1 for an age-period-cohort model in which the levels of the time effects are constrained in the first step leaving the ad hoc identification of the linear trends to the second step.

The first step constraints are affine of the type

(25) $$\begin{array}{c} C^{\prime }\theta_{C}=\psi , \end{array}$$

for known matrices *C* ∈ *R* ^*q*×(*q*−*q*_*C*_)^, *ψ* ∈ *R* ^*q*−*q*_*C*_^. The constrained time effect space is then

(26) $$\begin{array}{c} \Theta_{C}=\left(\theta_{C}\in \Theta :C^{\prime }\theta_{C}=\psi \right). \end{array}$$

Thereby the *q*-dimensional time effect space Θ is reduced to a *q*_*C*_-dimensional variation. The properties of this partially ad hoc identified parameter space depends on the rank of the matrix (*A*, *C*). If the number of constraints, *q* − *q*_*C*_, is at most equal to the number of unidentified components *q* − *p*, it is possible that (*A*, *C*) has full column rank. In that case the constraint implies a partial ad hoc identification without constraining the parameter space *M* of the statistical model. This is shown in Theorem 1; see also Section 5.4.1 for an example, while the proof is given in the Appendix. When (*A*, *C*) has reduced rank the parameter space *M* is also constrained; see Section 3.4.2 for a discussion.

**Theorem 1**

Suppose (*A*, *C*) has full column rank. Then the matrix *m* = *A*_⊥_′*C* ∈ *R* ^(*q*−*p*)×(*q*−*q*_*C*_)^ has full column rank and the constraint (25) does not constrain the canonical parameter *ξ* and the predictor *μ*. Hence, the predictor space remains of the form (2). The equivalence classes in Θ_*C*_ under the mapping *θ* ↦ *μ* = *XA*′*θ* are given by the group

(27) $$\begin{array}{c} g_{C}:\theta ⟼\theta +A_{⊥}m_{⊥}\zeta , \end{array}$$

for arbitrary *ζ* ∈ *R* ^*q*_*C*_−*p*^ where *m*_⊥_ ∈ *R* ^(*q*−*p*)×(*q*_*C*_−*p*)^ is the orthogonal complement of *m*. The maximal invariant remains *ξ* = *A*′*θ*.

The partial ad hoc identification by (25) implies that any time series analysis of the time effects has to happen relative to the constrained space Θ_*C*_ rather than the space Θ. This is awkward as discussed in Section 3.4 below. It is also considerably more complicated than working with the freely varying canonical parameter *ξ*; see Section 2.4.2.

### 3.4. Consequences of Ad Hoc Identification

In the following we will look closer at the consequences of working with the ad hoc identified time effect parameter *θ* in the context of a linear mortality model of the form *μ* = *Dθ*. We consider the consequences for plotting, hypothesis testing, and forecasting.

#### 3.4.1. Plots of Time Effects

In the mortality model (1) the time effect *θ* is the concatenation of age, period, and cohort effects. It seems natural to think of these individual time effects as time series and to plot them against time. As the time effect *θ* varies in the unrestricted space Θ = *R* ^*q*^ this maps the *q*-vector into unrestricted time series.

Estimates of the time effects are constructed by combining an estimate of *ξ* with an ad hoc chosen value for *λ* = *L*′*θ*, see (14). The resulting estimate ${\hat{\theta }}_{\lambda }$ is therefore constrained to the space Θ_*λ*_ ⊂ Θ. The interpretation of the estimate ${\hat{\theta }}_{\lambda }$ is therefore different from the interpretation of the original time effect *θ*. Distances on the spaces Θ and Θ_*λ*_ are judged differently and the variability of ${\hat{\theta }}_{\lambda }$ is deduced exclusively from $\hat{\xi }$ through (16). The time series components of ${\hat{\theta }}_{\lambda }$ are now restricted through *λ* = *L*′*θ*_*λ*_. Plots of the ${\hat{\theta }}_{\lambda }$-time series are therefore interpreted differently from the imagined plots of the original *θ*-time series and from the plots of the maximal invariant parameter *ξ* discussed in Section 2.4.2. Indeed, if one were to analyse the estimated ${\hat{\theta }}_{\lambda }$-time series statistically the linear constraint should be taken into account. This is a bit complicated as illustrated below, but it is the consequence of working with the ad hoc identified parameter *θ*_*λ*_ rather than the canonical parameter *ξ*.

Attempts to give intrinsic meaning to *λ* will be specific to the index set for the data set at hand. For instance, the requirement that the age effect should be zero on average does not carry over when looking at a subsample or when forecasting. It is not obvious that such an ad hoc identification is any more or less arbitrary than saying that, for instance, the first or the last age effect should have a particular value.

Adding confidence bands to a plot of ${\hat{\theta }}_{\lambda }$ is in itself not difficult. If $\hat{\xi }$ is asymptotically normal with mean *ξ* and variance Σ, then ${\hat{\theta }}_{\lambda }$ is asymptotically normal with mean *θ*_*λ*_ and variance *L*_⊥_(*A*′*L*_⊥_)^−1^Σ(*L*_⊥_′*A*)^−1^ *L*_⊥_′. This is a normal distribution on the space Θ_*λ*_. The interpretation of these standard errors will therefore be similar to that of ${\hat{\theta }}_{\lambda }$ itself.

Finally, it may be of interest to analyse the estimated ${\hat{\theta }}_{\lambda }$-time series statistically. Denote this time series by *x*_*λ*_. Its sample space is now Θ_*λ*_. A statistical model on Θ_*λ*_ can be built as follows. The starting point could be a time series model for unrestricted variables *x* on the sample space Θ. This gives a joint density for *x* ∈ Θ, which can be reduced by marginalisation to a density for *x*_*λ*_ ∈ Θ_*λ*_. Whether one is working with the unrestricted model for *x* ∈ Θ or the restricted model for *x*_*λ*_ ∈ Θ_*λ*_ inferences that are invariant to *g* must be based on those statistics of *x* or *x*_*λ*_ that are invariant to *g*. Thus, inferences must be based on the maximal invariant under *g*. For a general overview of invariant reduction see Cox and Hinkley [12, page 175f], whereas Nielsen [15] gives the argument in some detail for an autoregression with a linear trend.

#### 3.4.2. Hypothesis Testing

Having formulated the model in terms of time effects it may be of interest to test the hypothesis that one of these time effects is absent. No identification issues arise when the hypothesis is formulated as a restriction on the canonical parameter *ξ* as discussed in Section 2.4.3. But one has to be careful when formulating hypotheses in terms of the original time effect. See Sections 5.4.5, 5.5.3, and 5.5.4 for examples.

Affine hypotheses on the time effect are of the form

(28) $$\begin{array}{c} R^{\prime }\theta_{R}=\rho , \end{array}$$

for known matrices *R* ∈ *R* ^*q*×(*q*−*q*_*R*_)^, *ρ* ∈ *R* ^*q*−*q*_*R*_^. The constrained time effect space is then

(29) $$\begin{array}{c} \Theta_{R}=\left(\theta_{R}\in R^{q}:R^{\prime }\theta_{R}=\rho \right). \end{array}$$

To see how the restriction (28) restricts the predictor space *M* ⊂ *R* ^*n*^ recall that the predictor *μ* only depends on *θ* through *ξ* = *A*′*θ*. Thus, the analysis of the restriction (28) depends on the interplay between the matrices *A*, *R*.  Theorem A.3 in Appendix A.3 gives a general result to that effect. It shows that the hypothesis (28) restricts the predictor space *M* to a *p*_*R*_-dimensional affine subspace of *R* ^*n*^ in so far as it restricts the canonical parameter *ξ*. In particular, the degrees of freedom of the hypothesis, *p* − *p*_*R*_, may in general be different from the dimension reduction of the time effect parameter, *q* − *q*_*R*_. When this is the case the restriction (28) has an element of ad hoc identifying the time effect.

#### 3.4.3. Forecasts

Forecasts can be made by extrapolating the ad hoc identified time effects *θ*_*λ*_. Two researchers choosing different ad hoc identification schemes, but otherwise making the same analysis, may make different forecasts. This can be avoided if the extrapolation method is chosen with some care.

Following the linear approach outlined in Section 2.4.4 the predictor *μ* = *Dθ* = *XA*′*θ* is forecasted by extending the design *D* into

(30) $$\begin{array}{c} D^{h}=\left(\begin{array}{cc} D & 0\\ D_{1}^{h} & D_{2}^{h} \end{array}\right). \end{array}$$

Extrapolating the ad hoc identified *θ*_*λ*_ into a vector ${\left(\theta_{\lambda }^{\prime },{\tilde{\theta }}_{\lambda }^{\prime }\right)}^{\prime }$ then gives the forecast

(31) $$\begin{array}{c} \tilde{\mu }=\left(D_{1}^{h},D_{2}^{h}\right)\left(\begin{array}{c} \theta_{\lambda }\\ {\tilde{\theta }}_{\lambda } \end{array}\right)=D_{1}^{h}\theta_{\lambda }+D_{2}^{h}{\tilde{\theta }}_{\lambda }. \end{array}$$

Often both components *D*_1_^*h*^ *θ*_*λ*_ and $D_{2}^{h}{\tilde{\theta }}_{\lambda }$ depend on the ad hoc identification. Nonetheless, these dependencies of ad hoc identification may cancel each other so that the overall forecast $\tilde{\mu }$ is invariant to the ad hoc identification. Such invariance would seem desirable in most applications unless there is strong substantial reason for the ad hoc identification scheme. Necessary and sufficient conditions for invariance are presented for the age-period-cohort model in Section 5.4.3 and for a nonlinear model in Section 6.1.5.

In contrast, these considerations are redundant when working with the canonical parameter, *ξ*; see Section 2.4.4.

## 4. Bayesian Models and Random Effects Models

Mortality analysis is often carried out using either Bayesian methods or random effects methods. The mortality model is then altered through the introduction of a prior distribution on the parameters. One might think that the identification problems become less of an issue or even disappear. This is not the case since the Bayesian method and the random effects method is based on the mortality likelihood which only depends on the time effect *θ* through the maximal invariant parameter *ξ*. Thus, the identification challenges remain. The issue is that a prior on the unidentified part, say *λ*, of the time effect amounts to an ad hoc identification. Indeed, the conditional prior of *λ* given *ξ* is not updated by the mortality likelihood. A main difference is that a maximum likelihood analysis of the original mortality likelihood usually prompts the researcher when there is an identification issue, whereas both Bayesian methods and random effects methods allow computations to go through despite an identification issue.

In Section 4.1 it is seen that introduction of a conditional prior on *λ* given *ξ* is the Bayesian analogue of ad hoc identification. This leads to the same type of forecasting challenges as in the frequentist settings as is seen in Section 4.2. In Section 4.3 we show how the Bayesian identification issues transfer to random effects models.

### 4.1. Bayesian Estimation

For Bayesian and random effects models we formulate a likelihood and a prior. Thus, consider a likelihood *p*(*y* | *θ*) = *L*(*θ*; *y*). Replacing *θ* by *ξ*, *λ* the identification problem implies that

(32) $$\begin{array}{c} p\left(y\;|\;\xi ,\lambda \right)=p\left(y\;|\;\xi \right)\;\text{for}\;\;\text{all}\;\;\text{outcomes}\;\;y. \end{array}$$

The prior on *θ* is factorised as *p*(*θ*) = *p*(*ξ*, *λ*) = *p*(*ξ*)*p*(*λ* | *ξ*). In the case of Bayesian estimation the following result emerges.

**Theorem 2**

Suppose the likelihood satisfies (32). Then

(i) the predictive distribution does not depend on the conditional prior for *λ*:
  (33) $$\begin{array}{c} p\left(y\right)=\int p\left(y\;|\;\xi \right)p\left(\xi \right)d\xi ; \end{array}$$
(ii) the posterior satisfies
  (34) $$\begin{array}{c} p\left(\xi \;|\;y\right)=\frac{p\left(y\;|\;\xi \right)p\left(\xi \right)}{p\left(y\right)},\;\;p\left(\lambda \;|\;\xi ,y\right)=p\left(\lambda \;|\;\xi \right); \end{array}$$
(iii) the posterior means satisfy
  (35) $$\begin{array}{c} E\left(\xi \;|\;y\right)=\int \xi p\left(\xi \;|\;y\right)d\xi ,\\ E\left(\lambda \;|\;\xi ,y\right)=E\left(\lambda \;|\;\xi \right),\\ E\left(\lambda \;|\;y\right)=\int E\left(\lambda \;|\;\xi \right)p\left(\xi \;|\;y\right)d\xi . \end{array}$$

Theorem 2 shows that it suffices to give a prior to *ξ* and ignore *λ* as advocated in Section 2.4.5. Indeed the conditional prior for *λ* given *ξ* is not updated. Theorem 2 appears to be well-known; see Poirier [16, Proposition 2] or Smith [17, Section B].

Due to Theorem 2 the Bayesian analyst faces the complications outlined in Section 3.4. Indeed, suppose that two Bayesian researchers choose the same likelihood *p*(*x* | *ξ*, *λ*) = *p*(*x* | *ξ*) and the same prior *p*(*ξ*) for *ξ*, but different conditional priors for *λ* given *ξ*. Their marginal distributions for the data are identical, but any inferences regarding interpretation or forecasting will differ in so far as they involve the unidentified parameter *λ*. A Bayesian researcher should therefore be cautious with inference related to *λ*. There will of course be situations where the prior knowledge of *λ* given *ξ* is found to be of substantive relevance. In such situations it seems more fruitful to change the likelihood to include that information.

### 4.2. Forecasting

Bayesian forecasts involve integrating an extrapolative distribution. This can be done in two ways, either working exclusively with the identified, maximal invariant parameter *ξ* as in Section 2.4.4, or working with the time effect *θ* = (*ξ*, *λ*) as in Section 3.4.3.

#### 4.2.1. Forecasting Using the Maximal Invariant Parameter

Consider first the case where only the maximal invariant parameter *ξ* is used. In that case the forecast is computed by sampling from the posterior *p*(*ξ* | *y*) and then extrapolating $\tilde{\mu }$ using the sampled value *ξ* using some extrapolative methods, say $p(\tilde{\mu }|\xi ,y)$. In combination this gives the forecast

(36) $$\begin{array}{c} p\left(\tilde{\mu }\;|\;y\right)=\int p\left(\tilde{\mu }\;|\;\xi ,y\right)p\left(\xi \;|\;y\right)d\xi . \end{array}$$

#### 4.2.2. Forecasting Using the Ad Hoc Identified Time Effect

Consider now forecasts involving the full time effect *θ* = (*ξ*, *λ*). Theorem 2(ii) shows that the posterior satisfies *p*(*θ* | *y*) = *p*(*ξ* | *y*)*p*(*λ* | *ξ*). The distribution forecast with extrapolation $p(\tilde{\mu }|\xi ,\lambda ,y)$ is then

(37) $$\begin{array}{c} p\left(\tilde{\mu }\;|\;y\right)=\iint p\left(\tilde{\mu }\;|\;\xi ,\lambda ,y\right)p\left(\xi \;|\;y\right)p\left(\lambda \;|\;\xi \right)d\lambda \;d\xi . \end{array}$$

The concern is now as follows. Suppose a second researcher chooses the same extrapolative method, likelihood, and prior for *ξ*, but different conditional priors *p* ^†^(*λ* | *ξ*). In general, this will result in a different distribution forecast:

(38) $$\begin{array}{c} p^{†}\left(\tilde{\mu }\;|\;y\right)=\iint p\left(\tilde{\mu }\;|\;\xi ,\lambda ,y\right)p\left(\xi \;|\;y\right)p^{†}\left(\lambda \;|\;\xi \right)d\lambda \;d\xi . \end{array}$$

The question is then under which conditions will $p(\tilde{\mu }|y)=p^{†}(\tilde{\mu }|y)$ so that the distribution forecasts are invariant to the choice of conditional prior for *λ* given *ξ*? A sufficient condition is that the extrapolation method does not depend on *λ* so

(39) $$\begin{array}{c} p\left(\tilde{\mu }\;|\;\xi ,\lambda ,y\right)=p\left(\tilde{\mu }\;|\;\xi ,y\right). \end{array}$$

Condition (39) could alternatively be expressed as requiring that the forecast $p(\tilde{\mu }|\theta ,y)=p(\tilde{\mu }|\xi ,\lambda ,y)$ is invariant to the group *g* acting on the time effect space Θ so that $p(\tilde{\mu }|\xi ,\lambda ,y)=p\{\tilde{\mu }|\xi ,g(\lambda ),y\}$.

**Theorem 3**

Suppose that the likelihood satisfies (32) and the priors are probabilities. If the extrapolative distribution does not depend on *λ* so (39) holds; then the forecast distribution $p(\tilde{\mu }|y)$ computed in (37) is invariant to the choice of conditional prior for *λ* given *ξ*. The forecast then reduces to (36).

To summarise, the identification issues surrounding Bayesian analysis are similar to those outlined in the previous sections. Examples of the problems that can arise are discussed in Sections 5.4.4, 5.5.2, and 6.1.6. There are two solutions to the identification problem. The first is only to formulate a prior on *ξ*; see Section 2.4.5. Incidentally, this is what Bernardo and Smith [18, page 218] do in their discussion of the two-way analysis of variance, albeit without linking it to the considerations of Smith [17]. The prior *p*(*ξ*) can of course be constructed by formulating a prior on *θ* and then reduce it to a prior on *ξ* by marginalisation so *p*(*ξ*) = ∫*p*(*ξ*, *λ*)*dλ*. The other solution is to work with a prior on *θ* but avoid those parts of the posterior that depend on *λ*.

### 4.3. Random Effects Models

It is common to combine mortality models with a random effects approach, which effectively forms a new model. An example is given in Section 5.4.6. We consider the consequence of the lack of identification.

The random effect models are typically constructed as follows. Suppose the density of the data *y* given the time effects *θ* = (*ξ*, *λ*) is of the form *p*(*y* | *ξ*, *λ*) = *p*(*y* | *ξ*) as before; see (32). A prior *p*(*θ* | *ψ*) is chosen that now depends on a parameter *ψ*. The prior can be decomposed as *p*(*θ* | *ψ*) = *p*(*ξ* | *ψ*)*p*(*λ* | *ξ*, *ψ*). Theorem 2 implies that the density of the data *y* given *ψ* is

(40) $$\begin{array}{c} p\left(y\;|\;\psi \right)=\int p\left(y\;|\;\xi \right)p\left(\xi \;|\;\psi \right)d\xi . \end{array}$$

This in turn is used to form the random effects likelihood of *ψ* as

(41) $$\begin{array}{c} L_{\text{RE}}\left(\psi \;|\;y\right)=p\left(y\;|\;\psi \right). \end{array}$$

This, effectively, defines a new model. The random effects likelihood only depends on the prior *p*(*θ* | *ψ*) through *p*(*ξ* | *ψ*). Two researchers choosing the same prior *p*(*ξ* | *ψ*) but different conditional priors *p*(*λ* | *ξ*, *ψ*) will then get the same random effects likelihood and the same maximum likelihood estimator $\hat{\psi }$.

In mortality modelling it is common to go one step further and estimate the time effects *θ* through the mean of the posterior *p*(*θ* | *y*, *ψ*) evaluated at $\psi =\hat{\psi }$. Then the identification problem may show up. Theorem 2 shows that

(42) $$\begin{array}{c} p\left(\xi \;|\;\hat{\psi },y\right)=\frac{p\left(y\;|\;\xi \right)p\left(\xi \;|\;\hat{\psi }\right)}{p\left(y\;|\;\hat{\psi }\right)},\\ p\left(\lambda \;|\;\xi ,\hat{\psi },y\right)=p\left(\lambda \;|\;\xi ,\hat{\psi }\right), \end{array}$$

so that the prior for *ξ* is updated, while the conditional posterior for *λ* given *ξ* is not updated by the data. Thus, in general the estimate for *θ* is based, in part, on a prior which is not updated by the data.

## 5. Age-Period-Cohort Models

We will now apply the theoretical considerations to analyse the age-period-cohort model. The methodological literature on this model is large and the consequences of the above theory are wide ranging.

In Section 5.1 we present the age-period-cohort model along with the maximal invariant parameter. This maximal invariant parameter is also called the canonical parameter because the age-period-cohort model is usually implemented as an exponential family; see Section 2.4.1. When formulating the model we choose a notation matching the age-period-cohort literature rather than the reserving literature. At the same time the exposition takes it starting point in Kuang et al. [19], but the notation deviates.

The implementation of the canonical parameter depends on the type of data array. In Section 5.2 design matrices are given for age-cohort, age-period, and period-cohort data arrays. While they illustrate interesting differences in the structure for these data arrays, they also provide the basis for an immediate implementation via any generalised linear model software. The age-cohort model is expressed as a hypothesis of the age-period-cohort model in Section 5.3. Time effects and forecasting are considered in Section 5.4, while the two-sample age-period-cohort model is discussed in Section 5.5.

### 5.1. The Model and the Canonical Parameter

Here the age-period model is set up and a quite general identification result is reported.

Consider data *Y*_*ij*_ indexed by (*i*, *j*) ∈ *I* where *i* is the age and *j* is the period. The index set may be a rectangle given by *i* = 1,…, *I* and *j* = 1,…, *J* so that the cohort *k* = *I* − *i* + *j* runs from 1 to *K* = *I* + *J* − 1. More generally, the index set could be a generalized trapezoid where two corners are cut off the rectangle so that the cohort *k* runs from 1 + *h*_1_ to *I* + *J* − 1 − *h*_2_ for some *h*_1_, *h*_2_ ≥ 0. The class of generalized trapezoids includes the three types of Lexis diagrams discussed by Keiding [20]. We will return to those special cases below.

The statistical model is defined by the assumption that the variables *Y*_*ij*_ are independent with an exponential family distribution with predictor *μ*_*ij*_ given by

(43) $$\begin{array}{c} \mu_{ij}=\alpha_{i}+\beta_{j}+\gamma_{k}+\delta \;\text{for}\;\;i,j\in I. \end{array}$$

The time effect *θ* = (*α*_1_ …, *α*_*I*_, *β*_1_,…, *β*_*j*_, *γ*_*h*_1_+1_,…, *γ*_*I*+*J*−1−*h*_2__, *δ*)′ now varies in some time effect space Θ ∈ *R* ^*q*^ where *q* = *I* + *J* + *K* + 1 − *h*_1_ − *h*_2_.

The model (43) is of the form (1) discussed in Section 2. Specifically, the predictors *μ*_*ij*_ can be stacked in a vector *μ* of dimension *n* = dim⁡*I* and written as *μ* = *Dθ*. Thus, the parameter space for the model is of the form *M* = (*μ* ∈ *R* ^*n*^ : *μ* = *Dθ* for *θ* ∈ Θ) as outlined in (2). The mapping *θ* ↦ *μ* from Θ to *M* is surjective and the equivalence classes in the time effect space can be described by a group of transformations that are discussed in (8). This group can be represented as

(44) $$\begin{array}{c} g:\left(\begin{array}{c} \alpha_{i}\\ \beta_{j}\\ \gamma_{k}\\ \delta \end{array}\right)⟼\left(\begin{array}{c} \alpha_{i}+a+\left(i-1\right)d\\ \beta_{j}+b-\left(j-1\right)d\\ \gamma_{k}+c+\left(k-1\right)d\\ \delta -a-b-c-\left(I-1\right)d \end{array}\right)\;\text{for}\;\;\theta \in \Theta , \end{array}$$

for any *a*, *b*, *c*, and *d*. This is of the form (8) with *ζ* = (*a*,*b*,*c*,*d*)′ although the definition of the matrix *A* depends on the structure of the index set *I*.

**Figure 1:**
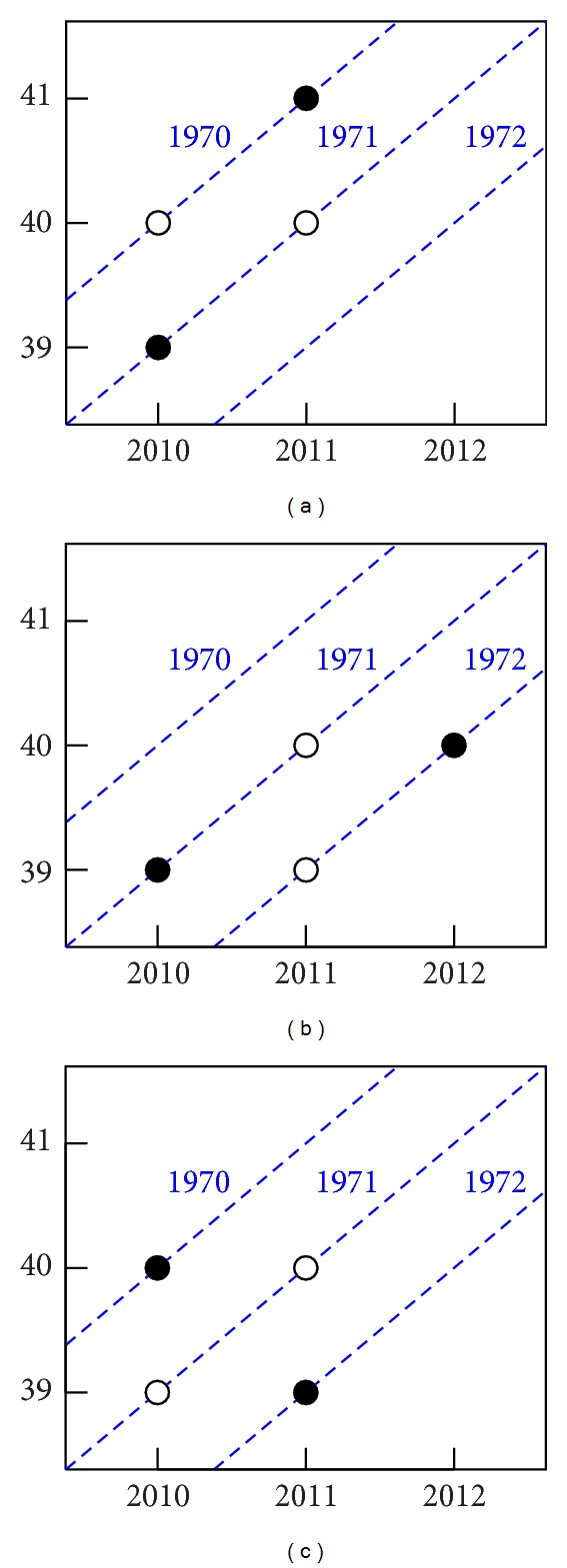
Illustration of interpretation of Δ^2^ *α*_41_, Δ^2^ *β*_2012_, and Δ^2^ *γ*_1972_.

A first clue for the canonical parametrisation is given by Fienberg and Mason [21] and Clayton and Schiffler [22] who pointed out that, on the multiplicative scale, ratios of relative risks are invariant. On the additive scale this amounts to looking at second differences, such as Δ^2^ *α*_*i*_ = *α*_*i*_ − 2*α*_*i*−1_ + *α*_*i*−2_. A graphical illustration of the double differences is given in Figure 1 (graphics were done using R 3.0.2, see R Development Core Team [23]), which is taken from Miranda et al. [24]. Panel (a) illustrates the interpretations of the formula for Δ^2^ *α*_*i*_ as follows. Consider the 1970 and 1971 cohorts. In 2010 these have ages 40 and 39, while in 2011 these have ages 41 and 40. Thus, Δ^2^ *α*_41_ represents the increase in mortality from ages 40 to 41 in 2011 relative to the increase from ages 39 to age 40 in 2010. An equivalent interpretation is that which represents the increase in mortality from ages 40 to 41 for the 1970 cohort relative to the increase from ages 39 to 40 for the 1971 cohort. In a similar way panels (b) and (c) illustrate the formulas for Δ^2^ *β*_2012_ and Δ^2^ *γ*_1972_.

Kuang et al. [19] introduces a parameter formed by these second differences as well as three entries of the predictor; that is,

(45) ξ=(μi1j1,μi2j2,μi3j3,Δ2α3,…,Δ2αI,Δ2β3,…,Δ2βJ,Δ2γh1+3,…,Δ2γK−h2).

The parameter *ξ* varies in the space *Ξ* = *R* ^*p*^ where *p* = *q* − 4. If the three points *μ*_*i*_1_*j*_1__, *μ*_*i*_2_*j*_2__, and *μ*_*i*_3_*j*_3__ are chosen not to be linearly related then they define the levels and the linear trends in the predictor. The formal condition is that a certain determinant defined from the indices is nonzero; that is,

(46) $$\begin{array}{c} i_{2}j_{3}-i_{3}j_{2}+i_{3}j_{1}-i_{1}j_{3}+i_{1}k_{2}-i_{2}k_{1}\neq 0. \end{array}$$

**Theorem 4 (see [19], [25, Corollary 2])**

Let *μ* satisfy (43). If the condition (46) is satisfied then the parameter *ξ* of (45) satisfies the following:

- *ξ* is a function of *θ* which is invariant to the group *g* in (44);
- *μ* is a function of *ξ*;
- the parametrisation of *μ* by *ξ* is exactly identified in that *ξ* ^†^ ≠ *ξ* ^‡^⇒*μ*(*ξ* ^†^) ≠ *μ*(*ξ* ^‡^).

Theorem 4 therefore shows that *ξ* varies freely in *Ξ* = *R* ^*p*^. Moreover, *ξ* is a maximal invariant of the mapping *m* from *θ* to *μ* under the transformations *g*. It should be noted that the choice of maximal invariant is not unique. Indeed, any bijective mapping of *ξ* can serve as maximal invariant. The choice of *ξ* is convenient since it becomes the canonical parameter in generalized linear models of the exponential family type.

In itself this theorem does not tell how to express the predictor *μ* in terms of the canonical parameter *ξ*. The link depends on the structure of the index set *I*. The above mentioned paper gives implicit expressions for generalized trapezoid index sets. In the following we report explicit expressions for the 3 principal Lexis diagrams.

### 5.2. Design Matrices for Lexis Diagrams

The link between the canonical parameter *ξ* and the predictor *μ* is analysed for the 3 principal Lexis diagrams. We start with age-cohort data arrays, which were the focus of attention in Kuang et al. [19]. Such arrays are easiest to analyse because all three time scales increase from the point where *i* = *j* = *k* = 1. As a consequence the results are relatively easier for these arrays.

#### 5.2.1. Age-Cohort Data Arrays

Age-cohort data arrays are rectangular in the age and cohort indices and given by

(47) $$\begin{array}{c} I_{\text{ac}}=\left\{\left(i,k\right):i=1,\ldots ,I,\;\;k=1,\ldots ,K\right\}. \end{array}$$

Consequently, the period index *j* = *i* + *k* − 1 varies over *j* = 1,…, *J* = *I* + *K* − 1. Keiding [20] refers to this Lexis diagram as the first principal set of death.

Age-cohort arrays are in particular used for reserving in general insurance. In that situation, only the triangle 1 ≤ *i*, *j*, *k* ≤ *I* is observed. The issue is to forecast the other triangle in the square 1 ≤ *i*, *k* ≤ *I*. In the reserving literature these triangles are referred to as the upper and lower triangles, since the cohort axis has reverse order. The two-factor age-cohort model for triangular age-cohort arrays is known as the chain-ladder model; see England and Verrall [26] for an overview. Zehnwirth [27] introduced an age-period-cohort model for such triangular arrays. The identification issue is analysed in detail in Kuang et al. [19, 25]. Subsequently, Kuang et al. [28] analysed the Poisson likelihood, while Kuang et al. [10] give an empirical analysis focusing on forecasting.

The age-period-cohort model for the age-cohort arrays is parametrised by

(48) $$\begin{array}{c} \mu_{ik}=\alpha_{i}+\beta_{i+k-1}+\gamma_{k}+\delta \;\text{for}\;\;i,k\in I_{\text{ac}}. \end{array}$$

The time effect *θ* = (*α*_1_,…,*α*_*I*_,*β*_1_,…,*β*_*J*_,*γ*_1_,…,*γ*_*K*_,*δ*)′ now varies in Θ = *R* ^2(*I*+*K*)^.

The design matrix linking the canonical parameter *ξ* in (45) and the predictor *μ* is essentially an identity linking the two parameters. A natural choice of the three levels points to the predictors that are *μ*_11_, *μ*_12_, and *μ*_21_. We then get the representation

(49) μik=μ11+(i−1)(μ21−μ11)+(k−1)(μ12−μ11) +∑l=3i∑h=3lΔ2αh+∑l=3j∑h=3lΔ2βh+∑l=3k∑h=3lΔ2γh,

with the convention that empty sums are zero, and recalling that second differences are defined as Δ^2^ *α*_*i*_ = *α*_*i*_ − 2*α*_*i*−1_ + *α*_*i*−2_ so that ∑_*h*=3_^*i*^Δ^2^ *α*_*h*_ = Δ*α*_*i*_ − Δ*α*_2_ and ∑_*l*=3_^*i*^∑_*h*=3_^*l*^Δ^2^ *α*_*h*_ = *α*_*i*_ − *α*_1_ − (*i* − 1)Δ*α*_2_.

The identity (49) is crucial to the understanding of the age-period-cohort model. It shows that the predictor has a single level expressed as *μ*_11_, which in turn satisfies *μ*_11_ = *α*_1_ + *β*_1_ + *γ*_1_ + *δ*. The level *μ*_11_ is therefore estimable, but the individual levels *α*_1_, *β*_1_, *γ*_1_, and *δ* are not identifiable from the model. Further, the model has two linear trends, here expressed with slopes *μ*_21_ − *μ*_11_ and *μ*_12_ − *μ*_11_ in terms of the age and cohort indices. These slopes can be expressed as *μ*_21_ − *μ*_11_ = Δ*α*_2_ + Δ*β*_2_ and *μ*_12_ − *μ*_11_ = Δ*β*_2_ + Δ*γ*_2_. They are estimable, but the individual slopes Δ*α*_2_, Δ*β*_2_, and Δ*γ*_2_ are not identifiable.

The design matrix now follows from the identity (49) so that the predictor satisfies *μ* = *Xξ*, where

(50) ξ=(μ11,μ21−μ11,μ12−μ11,Δ2α3,…,Δ2αI,Δ2β3,…,Δ2βJ,Δ2γ3,…,Δ2γK)′,

(51) Xik={1,(i−1),(k−1),h(i,3),…,h(i,I),h(j,3),…,h(j,J),h(k,3),…,h(k,K)}′,

where *ξ* ∈ *R* ^*p*^, where *p* = 2(*I* + *K* − 2) and *h*(*t*, *s*) = max⁡(*t* − *s* + 1,0).

The identification relies on Theorem 4, which can be specialised to age-cohort arrays as follows.

**Theorem 5 (see [19, Theorem 1])**

Let *μ* satisfy (48). The parameter *ξ* of (50) satisfies the following:

- *ξ* is a function of *θ* which is invariant to the group *g* in (44);
- *μ* is a function of *ξ*, because of (49);
- the parametrisation of *μ* by *ξ* is exactly identified in that *ξ* ^†^ ≠ *ξ* ^‡^⇒*μ*(*ξ* ^†^) ≠ *μ*(*ξ* ^‡^).

Theorem 5 in turn implies that the parameter *ξ* varies freely in *Ξ* = *R* ^*p*^, while the design matrix *X* given by (51) has full column rank. Originally, the more general Theorem 4 was proved as a corollary to Theorem 5.

#### 5.2.2. Age-Period Arrays

An age-period data array is rectangular in the age and cohort indices and given by

(52) $$\begin{array}{c} I_{\text{ap}}=\left\{\left(i,j\right):i=1,\ldots ,I,\;\;j=1,\ldots ,J\right\}. \end{array}$$

Consequently, the cohort index *k* = *j* − *i* + *I* varies over *k* = 1,…, *K* = *I* + *J* − 1. Keiding [20] refers to this Lexis diagram as the third principal set of death.

Age-period arrays are commonly used in epidemiology, in mortality analysis, and in sociology. The analysis of identification issue is largely similar to that of age-cohort arrays. However, the representation of the predictor *μ* in terms of *ξ* differs in an intriguing way, because the third time index, the cohort *k*, is the difference of the other two indices.

The age-period-cohort model for the age-period arrays is parametrised by

(53) $$\begin{array}{c} \mu_{ij}=\alpha_{i}+\beta_{j}+\gamma_{j-i+I}+\delta \;\text{for}\;\;i,j\in I_{\text{ap}}. \end{array}$$

The time effect *θ* = (*α*_1_,…,*α*_*I*_,*β*_1_,…,*β*_*J*_,*γ*_1_,…,*γ*_*K*_,*δ*)′ now varies in Θ = *R* ^2(*I*+*J*)^. A representation of the predictor *μ* in terms of the canonical parameter *ξ* is now

(54) μij=μI1+(i−I)(μI1−μI−1,1)+(j−1)(μI2−μI1) +∑l=iI−2∑h=lI−2Δ2αh+∑l=3j∑h=3lΔ2βh +∑l=3j−i+I∑h=3lΔ2γh+2.

The representation (54) differs from that of (49) in a subtle way. The three reference points for the levels of the predictor are chosen in the corner *i* = *I*, *j* = 1. From this corner period and cohort indices increase, while age decreases. Hence, the age double differences Δ^2^ *α*_*i*_ are now cumulated backwards. This phenomenon arises because the cohort index is the difference of the principal indices of age and period, whereas for the age-cohort array the period index is the sum of the principal indices of age and cohort. The predictor is now *μ* = *Xξ* where, with *h*(*t*, *s*) = max⁡(*t* − *s* + 1,0),

(55) ξ=(μI1,μI1−μI−1,1,μI2−μI1,Δ2α3,…,Δ2αI,Δ2β3,…,Δ2βJ,Δ2γ3,…,Δ2γK)′,

(56) Xij={1,i−I,j−1,h(1,i),…,h(I−2,i),(j,3),…,h(j,J),h(j−i+I,3),…,h(j−i+I,K)}′.

The identification relies on Theorem 4. It is specialised to age-period arrays as follows.

**Theorem 6 (see [24, Theorem 4.1])**

Let *μ* satisfy (53). The parameter *ξ* of (55) satisfies the following:

- *ξ* is a function of *θ* which is invariant to the group *g* in (44);
- *μ* is a function of *ξ*, because of (54);
- the parametrisation of *μ* by *ξ* is exactly identified in that *ξ* ^†^ ≠ *ξ* ^‡^⇒*μ*(*ξ* ^†^) ≠ *μ*(*ξ* ^‡^).

The group of transformations in (44) can be specialised as

(57) $$\begin{array}{c} g:\left(\begin{array}{c} \alpha_{i}\\ \beta_{j}\\ \gamma_{i-i+I}\\ \delta \end{array}\right)⟼\left(\begin{array}{c} \alpha_{i}+a+di\\ \beta_{j}+b-dj\\ \gamma_{j-i+I}+c+d\left(j-i+I\right)\\ \delta -a-b-c-dI \end{array}\right)\\ \text{for}\;\;\theta \in \Theta =R^{2(I+J)}; \end{array}$$

see, for instance, Carstensen [29]. This is of the form (8) with *ζ* = (*a*,*b*,*c*,*d*)′ and

(58) $$\begin{array}{c} A_{⊥}^{\prime }=\left(\begin{array}{c} \begin{array}{ccccccccccccc} 1 & 1 & \cdots & 1 & & & & & & & & & -1\\ & & & & 1 & 1 & \cdots & 1 & & & & & -1\\ & & & & & & & & 1 & 1 & \cdots & 1 & -1\\ 1 & 2 & \cdots & I & -1 & -2 & \cdots & -J & 1 & 2 & \cdots & K & -I \end{array} \end{array}\right). \end{array}$$

#### 5.2.3. Period-Cohort Arrays

An age-period data arrays is rectangular in the age and cohort indices and given by

(59) $$\begin{array}{c} I_{\text{pc}}=\left\{\left(j,k\right):j=1,\ldots ,J,\;\;k=1,\ldots ,K\right\}. \end{array}$$

Consequently, the age index *i* = *j* − *k* + *K* varies over *i* = 1,…, *I* = *J* + *K* − 1. Keiding [20] refers to this Lexis diagram as the second principal set of death. Age-period arrays are commonly used in prospective cohort studies in epidemiology and in sociology. The analysis is similar to that of age-period arrays when swapping the role of age and cohort.

The age-period-cohort model for the age-cohort arrays is parametrised by

(60) $$\begin{array}{c} \mu_{jk}=\alpha_{j-k+1}+\beta_{j}+\gamma_{k}+\delta \;\text{for}\;\;j,k\in I_{\text{ap}}. \end{array}$$

The time effect *θ* = (*α*_1_,…,*α*_*I*_,*β*_1_,…,*β*_*J*_,*γ*_1_,…,*γ*_*K*_,*δ*)′ now varies in Θ = *R* ^2(*J*+*K*)^. A representation of the predictor *μ* in terms of the canonical parameter *ξ* is now

(61) μjk=μ1K+(j−1)(μ2K−μ1K)+(k−K)(μ1K−μ1,K−1) +∑l=3j−k+1∑h=3lΔ2αh+∑l=3j∑h=3lΔ2βh +∑l=iK−2∑h=lK−2Δ2γh+2.

Thus, the canonical parameter and the design matrix are given by

(62) ξ=(μ1K,μ2K−μ1K,μ1K−μ1,K−1,Δ2α3,…,Δ2αI,Δ2β3,…,Δ2βJ,Δ2γ3,…,Δ2γK)′,

(63) Xjk={1,j−1,k−K,h(j−k+1,3),…,h(j−k+1,I),h(j,3),…,h(j,J),h(1,k),…,h(K−2,k)}′.

In parallel with Theorem 6 we then have the following identification result.

**Theorem 7**

Let *μ* satisfy (60). The parameter *ξ* of (62) satisfies the following:

- *ξ* is a function of *θ* which is invariant to the group *g* in (44);
- *μ* is a function of *ξ*, because of (61);
- the parametrisation of *μ* by *ξ* is exactly identified in that *ξ* ^†^ ≠ *ξ* ^‡^⇒*μ*(*ξ* ^†^) ≠ *μ*(*ξ* ^‡^).

### 5.3. Expressing the Age-Cohort Model as a Hypothesis

It is often of interest to test the absence of the period effect. An application to analysing asbestos related mortality can be found in Miranda et al. [24].

The hypothesis is that *β*_1_ = ⋯ = *β*_*J*_, when expressed in terms of the time effect parameters. The restricted model is given by, with *k* = *j* − *i* + *I*,

(64) $$\begin{array}{c} \mu_{ij}^{\text{ac}}=\alpha_{i}+\gamma_{j-i+I}+\delta \;\text{for}\;\;i,j\in I_{\text{ap}}. \end{array}$$

The identification problem simplifies to a question of determining the levels of *α*_*i*_ and *γ*_*k*_. Therefore the (log) relative risk parameters Δ*α*_*i*_ are identified as pointed out by Clayton and Schifflers [30]. In this model the cohort index is present and keeps the difference of the principal age and period indices. Therefore the representation of the predictor involves backward cumulated age differences as before but with a subtle change of sign, so that (54) reduces to

(65) $$\begin{array}{c} \mu_{ij}^{\text{ac}}=\mu_{I1}-\overset{I-1}{\underset{l=i}{\sum }}\Delta \alpha_{l+1}+\overset{k}{\underset{l=2}{\sum }}\Delta \gamma_{l}. \end{array}$$

As a consequence the canonical parameter and the design reduce to *μ*_*ij*_^ac^ = *X*_*ij*_^ac^ *ξ*_*ij*_^ac^, where

(66) $$\begin{array}{c} X_{ij}^{\text{ac}}=\left\{1,-1_{\left(1\geq i\right)},\ldots ,-1_{\left(I-1\geq i\right)},1_{\left(k\geq 2\right)},\ldots ,1_{\left(k\geq K\right)}\right\}, \end{array}$$

(67) $$\begin{array}{c} \xi^{\text{ac}}={\left(\mu_{I1},\Delta \alpha_{2},\ldots ,\Delta \alpha_{I},\Delta \gamma_{2},\ldots ,\Delta \gamma_{K}\right)}^{\prime }. \end{array}$$

Miranda et al. [24, Theorem 4.2] establish an identification result similar to Theorem 6.

The age-cohort model can also be formulated as a hypothesis on the maximal invariant *ξ* in the age-period-cohort model following Section 2.4.3. The period effects Δ^2^ *β*_*j*_ are set to zero through *H*′*ξ* = 0, where *H*′ = (0, *I*_*J*−2_, 0). Applying this to the expression for *ξ* in (55) gives

(68) ξH=H¯⊥′ξ=(μI1,ΔαI−Δγ2,Δγ2,Δ2α3,…,Δ2αI,Δ2γ3,…,Δ2γK),

since in the absence of period effects; then *μ*_*I*1_ − *μ*_*I*−1,1_ = Δ*α*_*I*_ − Δ*γ*_2_ and *μ*_*I*2_ − *μ*_*I*1_ = Δ*γ*_2_. The double differences cumulate to first differences through ∑_*i*=3_^*I*^Δ^2^ *α*_*i*_ = Δ*α*_*I*_ − Δ*α*_2_, so the above expression *ξ*_*H*_ is seen to be a linear transformation of *ξ* ^ac^ in (67). In other words the age-cohort model arises from the age-period-cohort model by restricting the maximal invariant parameter.

### 5.4. Working with the Time Effect

There is a large literature seeking to identify the original time effects *α*_*i*_, *β*_*j*_, and *γ*_*k*_ of the age-period-cohort model from the predictor. Here we look closer at some of those ad hoc identification proposals.

#### 5.4.1. Ad Hoc Identification of Levels

For the age-period-cohort model it is popular to impose ad hoc identifications in two steps of the type discussed in Section 3.3. Here the first step is concerned with the level of the time effects and the second step is concerned with the linear trend. Examples are given in Sections 5.4.2 and 5.5.4.

A common first step ad hoc identification is to require that

(69) $$\begin{array}{c} \overset{I}{\underset{i=1}{\sum }}\alpha_{i}=\overset{J}{\underset{j=1}{\sum }}\beta_{j}=\overset{K}{\underset{k=1}{\sum }}\gamma_{k}=0. \end{array}$$

This ad hoc identification is specific to the chosen data range. For instance, the constraint ∑_*i*=1_^*I*^ *α*_*i*_ = 0 is not easily transferable to a different data set drawn from the same population but with a different set of age groups. This aspect would have to be kept in mind if a substantive motivation was to be found for this constraint. Other ad hoc identification schemes such as *α*_*I*_ = *β*_*J*_ = *γ*_*K*_ = 0 have similar problems.

The constraint (69) is a special case of affine constraints of the form *C*′*θ*_*C*_ = *ψ* discussed in Section 3.3. The involved dimensions are *q* = 2(*I* + *J*) and *p* = *q* − 4, while the number of constrains is *q* − *q*_*C*_ = 3. The matrix *C*′ ∈ *R* ^(*q*−*q*_*C*_)×*q*^ is given by the top left {3 × (*q* − 1)}-block of *A*_⊥_′ in (58) padded with a column of zeros, while *ψ* ∈ *R* ^3^ is given by *ψ* = 0. Theorem 1 shows that *m* = *A*_⊥_′*C* ∈ *R* ^(*q*−*p*)×(*q*−*q*_*c*_)^ has full rank. Indeed, *m* and its orthogonal complement are given by

(70) $$\begin{array}{c} m=A_{⊥}^{\prime }C=\left(\begin{array}{ccc} I & 0 & 0\\ 0 & J & 0\\ 0 & 0 & K\\ I\overset{˘}{i} & -J\overset{˘}{j} & K\overset{˘}{k} \end{array}\right),\;\;m_{⊥}=\left(\begin{array}{c} -\overset{˘}{i}\\ \overset{˘}{j}\\ -\overset{˘}{k}\\ 1 \end{array}\right), \end{array}$$

where, for instance, $\overset{˘}{i}=I^{-1}\sum_{i=1}^{I}i=(I+1)/2$. Thus, the constrained group of equivalence classes (27) is

(71) gC:(αiβjγkδ)⟼{αi+d(i−i˘)βj−d(j−j˘)γk+d(k−k˘)δ}for  θ∈ΘC.

#### 5.4.2. Ad Hoc Identification of Slopes: The “Intrinsic” Estimator

The “intrinsic” estimator is a popular estimator in the sociology literature; see Yang et al. [4] and see also O'Brien [31, 32] and Fu et al. [33] for a recent discussion of its merits. It has its roots in a suggestion by Kupper et al. [34], with an early critique given by Holford [35].

The “intrinsic” estimator is defined in two steps. In the first step, the levels are identified by the ad hoc constraint (69). Three of the *θ*-coordinates are then dropped; that is *α*_*I*_, *β*_*J*_, and *γ*_*K*_ are dropped. In a second step the linear trend is ad hoc identified using a Moore-Penrose inverse as in (23).

We can analyse these steps using the developed framework. The first step identifies the levels by the ad hoc constraint (69), which is a constraint of the form *C*′*θ* = 0 for the *C* discussed in Section 5.4.1. This *θ* is defined on Θ_*C*_ which is a linear subspace with a dimension deficiency of 3. Introduce a selection matrix *S*_⊥_ ∈ *R* ^*q*×(*q*−3)^ that selects all coordinates of *θ* except *α*_*I*_, *β*_*J*_, and *γ*_*K*_. Thus *S*_⊥_ arises as a *q*-dimensional with 3 columns deleted corresponding to *α*_*I*_, *β*_*J*_, and *γ*_*K*_. This is chosen so that (*C*, *S*_⊥_) is invertible. Then *S*_⊥_′*θ* is freely varying in that *S*_⊥_′Θ_*C*_ = *R* ^*q*−3^. The skew projection identity *I*_*q*_ = *S*(*C*′*S*)^−1^ *C*′ + *C*_⊥_(*S*_⊥_′*C*_⊥_)^−1^ *S*_⊥_′ and the constraint *C*′*θ* = 0 then implies that *θ* = *C*_*S*_ *ϑ* where *C*_*S*⊥_ = *C*_⊥_(*S*_⊥_′*C*_⊥_)^−1^ and *ϑ* = *S*_⊥_′*θ* ∈ *R* ^*q*−3^. Note that while *C*_*S*⊥_ depends on *S*_⊥_ and *C*_⊥_, it does not depend on the normalisation of *C*_⊥_, since we can replace *C*_⊥_ by *C*_⊥_ *m* for arbitrary invertible matrices *m* ∈ *R* ^(*q*−3)×(*q*−3)^. This implies that *C*_*S*_ is a function of *S*_⊥_ and *C*. The predictor *μ* is now parametrised by *μ* = *XA*′*θ* = *XA*_*C*_′*ϑ* with *A*_*C*_′ = *A*′*C*_*S*_. This corresponds to equation 5 of Yang et al. [4] who use the notation *X* and *b* for *XA*_*C*_′ and *ϑ*, respectively.

In the second step the linear trend is ad hoc identified through a time effect parameter of the form (23) with *A*, *θ* replaced by *A*_*C*_, *ϑ* so that *θ*_ad.hoc_ = *C*_⊥_ *ϑ*_ad.hoc_ where *ϑ*_ad.hoc_ = *L*_⊥_(*A*_*C*_′*L*_⊥_)^−1^ *ξ* + (*A*_*C*_)_⊥_{*L*′(*A*_*C*_)_⊥_}^−1^ *λ* for some scalar *λ* and some matrix *L*_⊥_ ∈ *R* ^(*q*−3)×*p*^.

The “intrinsic” estimator is ad hoc identified through the choices *λ* = 0 and *L*_⊥_ = *A*_*C*_, while *C* is chosen by (69). It therefore estimates an “intrinsic” parameter:

(72) $$\begin{array}{c} \theta_{\text{intrinsic}}=C_{S⊥}C_{S⊥}^{\prime }A{\left(A^{\prime }C_{S⊥}C_{S⊥}^{\prime }A\right)}^{-1}\xi , \end{array}$$

which depends on the choices of *S*_⊥_′, *C*, and *A*_⊥_. However, since we can replace *C*_⊥_ by *C*_⊥_ *m* for arbitrary invertible matrices *m* ∈ *R* ^(*q*−3)×(*q*−3)^ without changing *θ*_intrinsic_ the expression *θ*_intrinsic_ does not depend on the normalisation of *C*_⊥_. The “intrinsic” parameter satisfies the following result.

**Theorem 8**

The “intrinsic” parameter is an injective mapping of the canonical parameter *ξ* ∈ *R* ^*p*^ into a *p* = *q* − 4 dimensional linear subspace Θ_intrinsic_ of Θ = *R* ^*q*^. The “intrinsic” time effect space is a *p*-dimensional linear subspace of *R* ^*q*^ of the form

(73) Θintrinsic ={θ∈Rq:θ=CS⊥CS⊥′A(A′CS⊥CS⊥′A)−1ξ  for  ξ∈Rp}, ={θ∈Rq:C′θ=0,  w′(C⊥′S⊥)(S⊥′C⊥)C¯⊥′θ=0},

where *w* ∈ *R* ^*q*−3^ is uniquely defined up to a scale by *w*′*C*_⊥_′*A* = 0.

Theorem 8 implies that the “intrinsic” parameter should be interpreted as an object varying in the linear subspace Θ_intrinsic_ rather than in the unrestricted time effect space Θ = *R* ^*q*^. As outlined in Section 3.4 this has consequences for the interpretation of plots of the time effects, hypothesis testing, and forecasts. A consequence of this argument is that different choices of *C*, *S*_⊥_, *L*, and *λ* would lead to other ad hoc identified parameters varying in other affine subspaces of Θ. In other words, the “intrinsic” estimator carries the cost of working with the somewhat complicated linear subspace Θ_intrinsic_. This effort may be worthwhile if the particular choice of *C*, *S*_⊥_, *L*, and *λ* can be made on substantive grounds.

#### 5.4.3. Forecasting

Forecasting of future mortality rates involves an extrapolation of the time parameters. In Section 2.4.4 it was argued that ad hoc identification may introduce an undesirable arbitrariness in the forecast. When working exclusively with the canonical parameter *ξ* this arbitrariness is avoided. It is, however, also possible to work with ad hoc identified time effects under specific circumstances that we characterise here for age-period arrays. This builds on the theory developed in Kuang et al. [25] for age-cohort data arrays.

In the context of an age-period data array *I*_ap_ it is often of interest to forecast *h* periods ahead. Suppose it is of interest to forecast the mortality at age *i* in period *J* + *h*, so that the cohort is *k* = *I* + *J* + *h* − *i*. This requires an extrapolation of the period effect. If the cohort index is sufficiently large, that is, *k* > *K*, then the cohort effect needs to be extrapolated too. Thus, there are two forecast index arrays of interest:

(74) $$\begin{array}{c} J_{\text{ap}}=\left\{\left(i,j\right):i=1,\ldots ,I;\;\;j=J+1,\ldots ,J+h;\;\;k\leq K\right\},\\ K_{\text{ap}}=\left\{\left(i,j\right):i=1,\ldots ,I;\;\;j=J+1,\ldots ,J+h;\;\;k>K\right\}. \end{array}$$

**Figure 2:**
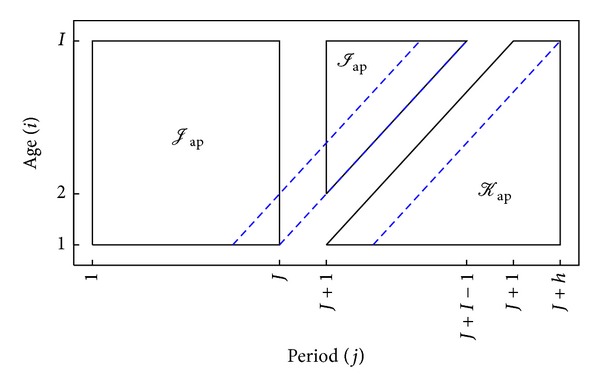
*I*_ap_ is the data array. *J*_ap,1_ is the forecast array where only period parameters need to be extrapolated. *J*_2_ is the forecast array where both period and cohort parameters need to be extrapolated. Cohorts are indicated by dashed lines.

Figure 2 illustrates these forecast index arrays.

Identification plays a role when extrapolating the estimates obtained on the data array *I*_ap_. The identification issues can be ignored if the investigator simply extrapolates Δ^2^ *β*_*j*_ and Δ^2^ *γ*_*k*_. In the context of ad hoc identified time effects arbitrary linear trends are introduced in the model. The forecast of the predictor *μ*_*i*,*J*+*h*_ is invariant to these if and only if the chosen extrapolation method for *β*_*j*_, *γ*_*k*_ preserves these linear trends so that they can cancel with the arbitrary linear trend in *α*_*i*_. The next result gives a precise formulation of this statement. It applies both to point forecasts and distribution forecasts.

**Theorem 9**

Consider the predictor *μ*_*ij*_ for *i*, *j* ∈ *I*_ap_ as given in (53). Suppose the time effects *α*_*i*_, *β*_*j*_, and *γ*_*k*_ are ad hoc identified. Consider the class of *h* periods-ahead forecasts over *J*_ap_ constructed as ${\tilde{\mu }}_{i,J+h}={\hat{\alpha }}_{i}+{\tilde{\beta }}_{J+h}+{\tilde{\gamma }}_{I+J+h-i}+\hat{\delta }$, where ${\tilde{\beta }}_{J+h}+{\tilde{\gamma }}_{I+J+h-i}$ is a function of the ad hoc identified estimate $\hat{\theta }$. Let *g* be the group (57). Invariance of the forecast ${\tilde{\mu }}_{i,J+h}$ with respect to the ad hoc identification is equivalent to either of the following:

(i) the extrapolation method for period and cohort effects is linear trend-preserving:
  (75) β~J+h(θ^)+γ~I+J+h−i(θ^) =[β~J+h{g(θ^)}−b+d(J+h)]  +[γ~I+J+h−i{g(θ^)}−c−d(I+J+h−i)]∀b,c,d∈R;
(ii) functions *f*_*β*_, *f*_*γ*_ exist so that with ${\hat{\xi }}_{\beta ,\gamma }={\left(\Delta^{2}{\hat{\beta }}_{3},\ldots ,\Delta^{2}{\hat{\beta }}_{J},\Delta^{2}{\hat{\gamma }}_{3},\ldots ,\Delta^{2}{\hat{\gamma }}_{K}\right)}^{\prime }$; then
  (76) β~J+h(θ^)+γ~I+J+h−i(θ^)={β^J+hΔβ^J+fβ(ξ^)} +{γ^K+(h−i+1)Δγ^K+fγ(ξ^)}.

To illustrate the use of Theorem 9 consider the extrapolation methods ${\tilde{\beta }}_{J+h}={\hat{\beta }}_{J}$ and $\Delta {\tilde{\beta }}_{J+h}=\Delta {\hat{\beta }}_{J}$. The first forecast is a random walk forecast and it is seen to violate (ii). The second forecast is a cumulated random walk and satisfies (ii). The reason is that *β*_*J*+*h*_ = *β*_*J*_ + ∑_*l*=1_^*h*^Δ*β*_*J*+*l*_. Since $\Delta {\tilde{\beta }}_{J+l}=\Delta {\hat{\beta }}_{J}$, then ${\tilde{\beta }}_{J+h}={\hat{\beta }}_{J}+h\Delta {\hat{\beta }}_{J}$. Further examples of forecasts that are linear trend-preserving as well as some which are not are given Kuang et al. [25, Table 1].

Kuang, Nielsen, and Nielsen [10] apply this to reserving data organised in an age-cohort array *I*_ac_ and discuss the issue of robustification of forecast with respect to structural breaks at the forecast origin. Miranda et al. [24] give an application to asbestos related mortality using an age-period array *I*_ap_.

#### 5.4.4. Bayesian Ad Hoc Identification Using a Dynamic Prior

A Bayesian ad hoc identification using a dynamic prior does not solve the identification problem as discussed in Section 4 and the same care has to be exercised to avoid the problems outlined in Section 3.4. Berzuini and Clayton [6] suggest such an ad hoc identification approach. On page 831 they write “*Identificability problems may be solved by imposing an arbitrary linear constraint on the log-linear trend components of age, period and cohort effects. Happily, such an arbitrary constraint has no effect on the predictions of the model*.” The previous analysis suggests that this is far from innocent.

The Berzuini-Clayton suggestion is to ad hoc identify the model (53) through

(77) $$\begin{array}{c} \alpha_{i}=\alpha_{1}+\alpha_{2}i+\overset{i}{\underset{l=3}{\sum }}\overset{l}{\underset{h=3}{\sum }}\Delta^{2}\alpha_{h},\\ \;\;\;\;\beta_{j}=\beta_{1}+\beta_{2}j+\overset{j}{\underset{l=3}{\sum }}\overset{l}{\underset{h=3}{\sum }}\Delta^{2}\beta_{h},\\ \gamma_{k}=\gamma_{1}+\gamma_{2}k+\overset{k}{\underset{l=3}{\sum }}\overset{l}{\underset{h=3}{\sum }}\Delta^{2}\gamma_{h},\\ \delta =0. \end{array}$$

A dynamic prior is chosen so that the double differences Δ^2^ *α*_*i*_, Δ^2^ *β*_*j*_, and Δ^2^ *γ*_*k*_ are independent zero mean normal with variances *ϕ* = (*σ*_*α*_^2^, *σ*_*β*_^2^, *σ*_*γ*_^2^) that have *χ* ^2^-type prior. The purpose of this is in part to facilitate extrapolations Δ^2^ *α*_*i*_, Δ^2^ *β*_*j*_, and Δ^2^ *γ*_*k*_ for *i* > *I*, *j* > *J*, and *k* > *K*, which is done through further draws from normal distributions. The level/trend effects *θ*_level_ = (*α*_1_, *α*_2_, *β*_1_, *β*_2_, *γ*_1_, *γ*_2_)′ have independent uniform priors on some large intervals.

We will analyse the Berzuini-Clayton model as applied to an age-period data array *I*_ap_. Decompose the canonical parameter *ξ* from (54) into two parts: the slope and level parameters, say *ξ*_*μ*_ = (*μ*_*I*1_, *μ*_*I*1_ − *μ*_*I*−1,1_, *μ*_*I*2_ − *μ*_*I*1_)′, and the collection of double differences, say *ξ*_Δ_. The assumed prior for *ξ*_Δ_ is a simple collection of independent normal distributions with variances *ϕ*. The assumed prior for *ξ*_*μ*_ is a linear combination of not only the independent uniform variables *θ*_level_, but also on *ξ*_Δ_, since the age double differences Δ^2^ *α*_*i*_ are cumulated backwards in (54), but forwards in (77). Thus, the prior for *ξ* = (*ξ*_*μ*_′, *ξ*_Δ_′)′ depends on the *θ*_level_ construction.

We get a hyper-parameter *λ*_hyper_ = (*λ*, *ϕ*), where *λ* is some three-dimensional ad hoc identified level/trend effect dependent on *θ*_level_, *ξ*_Δ_. We will argue that the ad hoc identified level/trend effect *λ* will wash out in the Berzuini-Clayton model. However, the level/trend parameter *ξ*_*μ*_ is a function of the *θ*_level_ construction that is tailored to the ad hoc identification. That construction remains in the analysis.

In the presentation of the posterior Berzuini and Clayton are careful only to consider the double differences *ξ*_Δ_ and stay clear of the ad hoc identified level/trend effect *θ*_level_. Theorem 2 yields the posterior *p*(*ξ* | *y*) = *p*(*y* | *ξ*)*p*(*ξ*)/*p*(*y*). Thus, the marginal posterior for the double differences is *p*(*ξ*_Δ_ | *y*) = ∫*p*(*y* | *ξ*_Δ_, *ξ*_*μ*_)*p*(*ξ*_Δ_, *ξ*_*μ*_)*dξ*_*μ*_/*p*(*y*). This links *ξ*_Δ_ to *ξ*_*μ*_ and in turn to the *θ*_level_ construction.

The extrapolative method is based on double differences so it only depends on *λ*_hyper_ through *ϕ* due to Theorem 9 and the subsequent discussion. Thus, the extrapolative method is of the form $p(\tilde{\mu }|\xi ,\lambda_{\text{hyper}},y)=p(\tilde{\mu }|\xi ,\phi ,y)$. By construction it does not reduce to $p(\tilde{\mu }|\xi ,y)$ so that condition (39) for Theorem 3 is not satisfied. The distribution forecast is of the form

(78) $$\begin{array}{c} p\left(\tilde{\mu }\;|\;y\right)=\iint p\left(\tilde{\mu }\;|\;\xi ,\phi ,y\right)p\left(\xi \;|\;y\right)p\left(\phi \;|\;\xi \right)d\phi \;\;d\xi , \end{array}$$

which, apart from depending on the *θ*_level_ construction, also depends on the conditional prior *p*(*ϕ* | *ξ*), which is not updated by the likelihood.

In summary, it appears that the Berzuini-Clayton analysis depends on the *θ*_level_ construction as well as the conditional prior *p*(*ϕ* | *ξ*). The dependence on the *θ*_level_ construction could of course be addressed by introducing priors directly on *ξ*_*μ*_, which in turn would be updated by the likelihood. Since the conditional prior *p*(*ϕ* | *ξ*) cannot be updated by the likelihood that its sole justification rests on the substantial context.

#### 5.4.5. A Functional Form Hypothesis

It is instructive to consider functional form restrictions on the time effects. Such hypotheses can be analysed using the results outlined in Section 3.4.2. As an example restrict the age effect to be quadratic in a similar way to Yang and Land [5] so that

(79) $$\begin{array}{c} \alpha_{i}=\sigma_{0}+\sigma_{1}i+\sigma_{2}i^{2}\;\text{for}\;\;i=1,\ldots ,I. \end{array}$$

This restriction on the time effect can be analysed by writing it on the form *R*′*θ* = *ρ*, see (28), and then applying Theorem A.3. Alternatively, in this particular case, we can show that the restriction actually only affects the ad hoc identified time effect through the canonical parameter, so a simpler analysis can be made.

A quadratic polynomial has constant second order derivative. Therefore the restriction (79) implies

(80) $$\begin{array}{c} \Delta^{2}\alpha_{i}=2\sigma_{2}\;\text{for}\;\;i=3,\ldots ,I. \end{array}$$

This expression has one free parameter. Thus, it is useful to consider the third order difference:

(81) $$\begin{array}{c} \Delta^{3}\alpha_{i}=\Delta^{2}\alpha_{i}-\Delta^{2}\alpha_{i-1}=0\;\text{for}\;\;i=4,\ldots ,I. \end{array}$$

This gives *I* − 3 linear restrictions on the canonical parameter. The age time effect *α*_*i*_ then has three remaining parameters, say *α*_1_, *α*_2_, and *α*_3_. These are freely varying since the parameters *σ*_0_, *σ*_1_, and *σ*_2_ are freely varying.

If the constraint is imposed directly on the canonical parameter, the restricted model is a regular exponential family with the advantages outlined in Section 2.4. However, if the analysis is done with the time effect the levels and trend will have to be ad hoc identified while bearing in mind the issues discussed above.

#### 5.4.6. The “Hierarchical Age-Period Cohort Regression Model”

In some cases a random effects approach can be used to get an overview of the many parameters of the age-period model. When applied to the time effects this implies an ad hoc identification. An example is the “hierarchical age-period cohort regression model” by Yang and Land [5]. In that paper the age effect is given a quadratic structure, but that does not have to be the case. The model is then given by

(82) $$\begin{array}{c} \alpha_{i}=\sigma_{0}+\sigma_{1}i+\sigma_{2}i^{2},\;\;\beta_{j}\overset{D}{=}N\left(0,\sigma_{\beta }^{2}\right),\\ \gamma_{k}\overset{D}{=}N\left(0,\sigma_{\gamma }^{2}\right),\;\;\delta =0. \end{array}$$

Since random effects are only introduced for some of the time effects, the analysis of Section 4.3 has to modified in a similar way to the analysis in Section 5.4.4.

From (80) it is seen that the model restricts Δ^2^ *α*_*i*_ = 2*σ*_2_. Thus, divide the canonical parameter *ξ* into three elements: the slope and level parameters, say *ξ*_*μ*_ = (*μ*_*I*1_, *μ*_*I*1_ − *μ*_*I*−1,1_, *μ*_*I*2_ − *μ*_*I*1_)′, the age-double differences *ξ*_*α*_ = (Δ^2^ *α*_3_,…, Δ^2^ *α*_*I*_), and the remaining double differences *ξ*_*β*,*γ*_. Here *ξ*_*α*_ is restricted by the hypothesis 2*σ*_2_ and *ξ*_*β*,*γ*_ is linear function of the normal random effects, while *ξ*_*μ*_ is a three-dimensional linear function of *σ*_2_ and of the six-dimensional object *ν* = (*σ*_0_, *σ*_1_, *β*_1_, *β*_2_, *γ*_1_, *γ*_2_)′. This leaves a three-dimensional ad hoc identified level/slope parameter *λ* which is also a function of *ν* but not entering the likelihood. Let *ψ* = (*σ*_0_, *σ*_1_, *σ*_2_, *σ*_*β*_^2^, *σ*_*γ*_^2^).

The random effects likelihood are constructed in three steps. First, we have the usual age-period-cohort likelihood *p*(*y* | *ξ*). Secondly, the random effects distribution for *ξ*_*μ*_, *ξ*_*β*,*γ*_, and *λ* is multivariate normal, while *ξ*_*α*_ is deterministic function of *ψ*. Thus, decompose the prior as *p*(*ξ*_*μ*_, *ξ*_*β*,*γ*_, *λ* | *ψ*) = *p*(*ξ*_*μ*_, *ξ*_*β*,*γ*_ | *ψ*)*p*(*λ* | *ξ*_*μ*_, *ξ*_*β*,*γ*_, *ψ*). Thirdly, following Section 4.3 the random effects likelihood will not depend on *p*(*λ* | *ξ*_*μ*_, *ξ*_*β*,*γ*_, *ψ*) and it is given by

(83) p(y ∣ ψ) =∫p(y ∣ ξα,ξμ,ξβ,γ)p(ξα,ξμ,ξβ,γ ∣ ψ)d(ξμ,ξβ,γ).

The prior *p*(*λ* | *ξ*_*μ*_, *ξ*_*β*,*γ*_, *ψ*) is not updated by the data. Plots and inferences based on the posterior *p*(*θ* | *ψ*, *y*) will then suffer from the ad hoc identification issues outlined in Section 3.4.

### 5.5. A Two-Sample Age-Period-Cohort Model

When confronted with two samples for women and for men it may be of interest to apply the age-period-cohort model (43) to each of the samples and impose that some of the time effects are the same across samples. The models for samples *r* = 1,2 are

(84) $$\begin{array}{c} \mu_{ijr}=\alpha_{ir}+\beta_{jr}+\gamma_{kr}+\delta_{r}\;\text{for}\;\;i,j\in I,\;\;r=1,2. \end{array}$$

The time effect *θ* = (…, *α*_*ir*_, *β*_*jr*_, *γ*_*kr*_, *δ*_*r*_,…)′ now varies in Θ = *R* ^*q*^ where *q* = 4(*I* + *J*).

#### 5.5.1. Analysis of the Unrestricted Two-Sample Model

The unrestricted two-sample model is simply analysed as two copies of the one sample model of Section 5.1. The time effects of each copy are only defined up to linear trends. The group of transformations characterizing the identification problem combines two copies of the one sample group (44). The maximal invariant parameter is *ξ* = (*ξ*_1_′, *ξ*_2_′)′ ∈ *R* ^*p*^ where *p* = 4(*I* + *J* − 2) and each of *ξ*_*r*_ are of the form (45). The benefits of Section 2 hold when working with that parameter.

#### 5.5.2. Bayesian Ad Hoc Identification Using a Dynamic Model

An application of the unrestricted two-sample model can be found in Cairns et al. [36]. The two samples are the population of England and Wales and the subpopulation of assured lives, so the substantive question is whether there is a selection effect for the assured lives. A Bayesian model with dynamic prior is used. It shares some features with the Berzuini and Clayton [6] model discussed in Section 5.4.4 although the details of the ad hoc identification of the levels and slopes are slightly different. When it comes to forecasting the extrapolative method appears to depend on the ad hoc identified parameter as well as the hyperparameters. This complicates the analysis of the forecast relatively the discussion in Section 5.4.4.

#### 5.5.3. The Hypothesis of Common Period Parameters

The two-sample model allows the possibility for adding cross-sample restrictions on the parameters. As an example we consider the hypothesis of common period parameters.

Working with the canonical parameter the hypothesis is

(85) $$\begin{array}{c} \Delta^{2}\beta_{i1}=\Delta^{2}\beta_{i2}\;\text{for}\;\;j=3,\ldots ,J. \end{array}$$

This is a simple linear restriction as that discussed in Section 2.4.3. It is readily seen that the degrees of freedom of the hypothesis are *p* − *p*_*H*_ = *J* − 2 so the dimension of the restricted model is *p*_*H*_ = 4*I* + 3*J* − 6. The canonical parameter under the hypothesis is then

(86) ξH=(…,μ11r,μ21r−μ11r,μ12r−μ11r,Δ2αir,Δ2βj,Δ2γkr,…)′.

The same result arises when writing the hypothesis in terms of time effects so that

(87) $$\begin{array}{c} \beta_{j1}=\beta_{j2}\;\text{for}\;\;j=1,\ldots ,J. \end{array}$$

Such hypotheses on the time effect were discussed in Section 3.4.2. It can be analysed using the general result in Theorem A.3. However, we will take the simpler route of arguing that this only restricts the canonical parameter given a hypothesis of the type (85). The argument relies on noting that analysing the restriction for the predictors *μ*_*ij*1_ and *μ*_*ij*2_ is equivalent to analysing the restriction for the predictors *μ*_*ij*1_ and *μ*_*ij*2_ − *μ*_*ij*1_, where the cross-sample differenced predictor is of the form

(88) μij2−μij1=(αi2−αi1)+(βj2−βj1)+(γk2−γk1) +(δ2−δ1).

Now, the restricted model for the cross-sample differenced predictor *μ*_*ij*2_ − *μ*_*ij*1_ is an age-cohort model:

(89) $$\begin{array}{c} \mu_{ij2}-\mu_{ij1}=\left(\alpha_{i2}-\alpha_{i1}\right)+\left(\gamma_{k2}-\gamma_{k1}\right)+\left(\delta_{2}-\delta_{1}\right). \end{array}$$

Following the analysis of Section 5.3 the (87) therefore implies the *J* − 2 linear restrictions given by (85). At the same time the predictor for the first sample *μ*_*ij*1_ is left unrestricted by (87). In summary, the restrictions (85) and (87) are equivalent.

The restriction has an interesting implication for the interpretation of the involved double differences. For the unrestricted model it was found that only plain double differences, such as Δ^2^ *α*_*jr*_, are identified. Under the restriction the cross-sample differenced predictor is of age-cohort form (89) so also the cross-double differences Δ(*α*_*i*2_ − *α*_*i*1_) and Δ(*γ*_*k*2_ − *γ*_*k*1_) are identified.

#### 5.5.4. Step-Wise Ad Hoc Identification under the Hypothesis

The analysis of Riebler and Held [7] finds that the difference *α*_*i*2_ − *α*_*i*1_ is identified under the hypothesis (85). This is not consistent with the above analysis showing that the cross-sample differenced predictor is an age-cohort model under the hypothesis, for which levels such as *α*_*i*2_ − *α*_*i*1_ are identified.

The apparent difference comes about because Riebler and Held follow a step-wise identification approach along the lines of Sections 3.3 and 5.4.1. In a first step the time effects *α*_*ir*_, *β*_*jr*_, and *γ*_*kr*_ are constrained to have zero-sums as in (69). In a second step the slopes are ad hoc identified using a Bayesian approach similar to that of Berzuini and Clayton [6]; see Sections 4 and 5.4.4 for a discussion of the consequences.

The identification in the first step implies that *α*_*i*2_ − *α*_*i*1_ has a zero sum. Under the hypothesis (85) this is exactly what is needed to ad hoc identify the levels in the age-cohort model (89). In other words a different level identification in the first step leads to a different level for the difference *α*_*i*2_ − *α*_*i*1_.

## 6. Models with Nonlinear Parametrisations

Some additional issues arise when looking at models with nonlinear parametrisations. A prominent example is the mortality model proposed by Lee and Carter [3] and which is the current benchmark in mortality studies done by government agencies and pension funds. For this model the time effect space Θ has a nondifferentiability which can actually be avoided by working directly with the parameter space *M*.

We analyze the Lee-Carter model in Section 6.1. In Section 6.2 we turn to a two-sample problem where some additional difficulties can arise when forecasting.

### 6.1. The Lee-Carter Model

The mortality model proposed by Lee and Carter [3] has predictor of the form

(90) $$\begin{array}{c} \mu_{ij}=\alpha_{i}+\beta_{i}\kappa_{j}\;\text{for}\;\;i,j\in I_{\text{ap}}. \end{array}$$

The time effects *θ* = (*α*_1_,…, *α*_*I*_, *β*_1_,…, *β*_*I*_, *κ*_1_,…, *κ*_*J*_) vary in Θ = *R* ^2*I*+*J*^.

Lee and Carter pointed towards two identification issues of the model. If *α*, *β*, and *κ* are one solution to (90), then *α* − *βc*, *β*, *κ* + *c* is also a solution for any scalar *c*, just as *α*, *β*/*d*, and *κd* are a solution for any *d* ≠ 0. Consequently, they proposed the ad hoc identification:

(91) $$\begin{array}{c} \sum_{i}\beta_{i}=1,\;\;\sum_{j}\kappa_{j}=0. \end{array}$$

This is, however, not the full story about the identification issues. To get at this we follow the outline from the linear parametrised models and start by finding the parameter space for the predictor *μ*.

#### 6.1.1. The Parameter Space

We start by finding the predictor space *M*. Write the model in matrix form. Let $\underline{\mu }$ denote the *I* × *J*-matrix of *μ*_*ij*_. Then

(92) $$\begin{array}{c} \underline{\mu }=\alpha \iota^{\prime }+\beta \kappa^{\prime }, \end{array}$$

where *α*, *β*, and *κ* are vectors concatenating *α*_*i*_, *β*_*i*_, and *κ*_*j*_ and where *ι* = (1,…, 1)′ ∈ *R* ^*J*^. Postmultiply by the projection identity $I_{J}=\bar{\iota }\iota^{\prime }+{\bar{\iota }}_{⊥}\iota_{⊥}^{\prime }$ to get

(93) $$\begin{array}{c} \underline{\mu }=\alpha \iota^{\prime }+\beta \kappa^{\prime }\left(\bar{\iota }\iota^{\prime }+{\bar{\iota }}_{⊥}\iota_{⊥}^{\prime }\right)=\left(\alpha +\beta \kappa^{\prime }\bar{\iota }\right)\iota^{\prime }+\beta \left(\kappa^{\prime }{\bar{\iota }}_{⊥}\right)\iota_{⊥}^{\prime }, \end{array}$$

where the orthogonal complement *ι*_⊥_ can be chosen so that *ι*_⊥_′*κ* = (Δ*κ*_2_,…, Δ*κ*_*J*_)′ but could also be chosen otherwise. Equation (93) shows that the model is composed of two matrices with rank one. Thus, the parameter space is given by

(94) M={μ_∈RI×J:μ_=γι′+δι⊥′for  (γ,δ)∈RI×RI×(J−1)so  rank⁡(δ)≤1}.

Note that *M* does not depend on the normalisation of *ι*_⊥_ since *δ* is freely varying. The space *M* is a manifold since the space of matrices *δ* with an upper bound to the rank is a manifold as opposed to the space where *δ* has rank of unity. This space can be parametrised parsimoniously by

(95) $$\begin{array}{c} \xi =\left(\gamma ,\delta \right)\;\text{where}\;\;\gamma =\alpha +\beta \kappa^{\prime }\bar{\iota },\;\delta =\beta \kappa^{\prime }{\bar{\iota }}_{⊥}, \end{array}$$

varying in the manifold

(96) $$\begin{array}{c} \Xi =\left\{\left(\gamma ,\delta \right)\in R^{I}\times R^{I\times (J-1)}:\mathrm{rank}\left(\delta \right)\leq 1\right\}. \end{array}$$

The *ξ* is the candidate for the maximal invariant describing the equivalence classes of the mapping from the time effect *θ* to the predictor *μ*.

The next step is to analyse the time effect space Θ. It is convenient to decompose *M* into two disjoint sets depending on the rank of *δ*. These sets are

(97)  M1={μ_∈RI×J:μ_=γι′+δι⊥′  for  (γ,δ)∈RI×RI×(J−1)so  rank⁡(δ)=1}, M0=(μ_∈RI×J:μ_=γι′  for  γ∈RI).

Correspondingly, the time effect space Θ can be decomposed into two disjoint sets:

(98) $$\begin{array}{c} \Theta_{1}=\left(\theta \in \Theta :\exists i,j\;\;\text{so}\;\;\beta_{i}\kappa_{j}\neq \beta_{i}\kappa_{J}\right),\\ \Theta_{0}=\left(\theta \in \Theta :\forall i,j\;\;\text{so}\;\;\beta_{i}\kappa_{j}=\beta_{i}\kappa_{J}\right). \end{array}$$

Note that *δ* = 0 if and only if *θ* ∈ Θ_0_. Consider first the time effect space Θ_1_, which is implicitly what Lee and Carter had in mind. The mapping *θ* ↦ *μ* on Θ_1_ to *M* is invariant to the group of transformations:

(99) $$\begin{array}{c} g_{1}:\left(\begin{array}{c} \alpha_{i}\\ \beta_{i}\\ \kappa_{j} \end{array}\right)⟼\left(\begin{array}{c} \alpha_{i}+\beta_{i}c\\ \frac{\beta_{i}}{d}\\ \left(\kappa_{j}-c\right)d \end{array}\right), \end{array}$$

acting on Θ_1_ for all *c* ∈ *R* and all *d* ≠ 0. The parameter *ξ* = (*γ*′, *δ*′)′ is invariant under *g*_1_ acting on Θ_1_. Now, consider the space Θ_0_ with deficient rank. Then *α*_*i*_, *β*_*i*_, and *κ*_*j*_ map into *α*_*i*_ + *φ*_*i*_ where *φ*_*i*_ = *β*_*i*_ *κ*_*j*_ is constant in *j*, so that $\delta =\beta \kappa^{\prime }{\bar{\iota }}_{⊥}=0$. This mapping is invariant to the group of transformations:

(100) $$\begin{array}{c} g_{0}:\left(\begin{array}{c} \alpha_{i}\\ \beta_{i}\kappa_{j} \end{array}\right)⟼\left(\begin{array}{c} \alpha_{i}+a_{i}\\ \beta_{i}\kappa_{j}-a_{i} \end{array}\right), \end{array}$$

acting on Θ_0_ for all (*a*_1_,…, *a*_*I*_)′ ∈ *R* ^*I*^.

**Theorem 10**

Let $\underline{\mu }\in M$. The parameter *ξ* ∈ *Ξ* of (95) satisfies the following:

- *ξ* is a function of *θ* ∈ Θ which is invariant to the groups *g*_0_, *g*_1_ in (99) and (100);
- $\underline{\mu }$ is a function of *ξ*;
- the parametrisation of $\underline{\mu }$ by *ξ* is exactly identified in the sense that $\xi^{†}\neq \xi^{‡}\Rightarrow \underline{\mu }(\xi^{†})\neq \underline{\mu }(\xi^{‡})$.

Theorem 10 shows that *ξ* varies freely on the space *Ξ* and it gives a unique parametrisation of *μ*. As a function of *θ* it is invariant to *g*_0_, *g*_1_; hence it is a maximal invariant.

It is interesting to compare the properties of the spaces *M*, *Ξ*, and Θ. The spaces *M* and *Ξ* are spaces of matrices with deficient rank. These are smooth spaces, but they are not vector spaces since the sum of matrices with rank one may have rank larger than one. In contrast Θ is a vector space. The mapping from Θ to *M* will inevitably be nondifferentiable. This nondifferentiability is avoided by working directly with *M*. Likewise, in a Bayesian setting it would seem more difficult to introduce a meaningful prior of Θ with its nondifferentiability than on *M*.

#### 6.1.2. Maximum Likelihood Estimation

The maximum likelihood estimator for *ξ* can be derived analytically in the normal case.

Consider a situation where the data array is of age-period form so *Y*_*ij*_ for (*i*, *j*) ∈ *I*_ap_. Suppose *Y*_*ij*_ are independent normal with mean *μ*_*ij*_ and variance *σ* ^2^. Organise the data in a matrix $\underline{Y}$. Then the log likelihood is of the form

(101) $$\begin{array}{c} l\left(\underline{\mu },\sigma^{2};\underline{Y}\right)=-\frac{IJ}{2}\log\left(2\pi \sigma^{2}\right)-\frac{1}{2\sigma^{2}}\mathrm{tr}\left\{\left(\underline{Y}-\underline{\mu }\right){\left(\underline{Y}-\underline{\mu }\right)}^{\prime }\right\}. \end{array}$$

The maximum likelihood estimator is of the following form. Subsequently, this is related to the estimator suggested by Lee and Carter.

**Theorem 11**

For a normal age-period array parametrised by (94) the maximum likelihood estimators are

(102) $$\begin{array}{c} \hat{\gamma }=\underline{Y}\iota {\left(\iota^{\prime }\iota \right)}^{-1},\;\;\hat{\delta }=\left[{\text{svd}}_{1}\left\{\underline{Y}\iota_{⊥}{\left(\iota_{⊥}^{\prime }\iota_{⊥}\right)}^{-1}\iota_{⊥}^{\prime }\right\}\right]\iota_{⊥}{\left(\iota_{⊥}^{\prime }\iota_{⊥}\right)}^{-1}, \end{array}$$

where svd_1_(·) is the singular value decomposition truncated to one factor.

Thus, *γ* is estimated by the row-averages of the data matrix, while *δ* is estimated by the singular value decomposition of the row-wise demeaned data matrix.

#### 6.1.3. Estimation of Ad Hoc Identified Time Effects

The ad hoc identification (91) gives a time effect *θ*_*λ*_ varying in a 2*I* + *J* − 2 dimensional affine subspace of Θ = *R* ^2*I*+*J*^. The ad hoc identified *θ*_*λ*_ can now be expressed in terms of the maximal invariant parameter *ξ* using (95). In the case where *δ* ≠ 0 then it has singular value decomposition *δ* = *δ*_*L*_ *δ*_*S*_ *δ*_*R*_′ for two vectors *δ*_*L*_ ∈ *R* ^*I*^ and *δ*_*R*_ ∈ *R* ^*J*−1^ so *δ*_*L*_′*δ*_*L*_ = 1 and *δ*_*R*_ *δ*_*R*_′ = 1, while *δ*_*S*_ > 0 is a positive scale. The ad hoc identification of Lee and Carter then gives

(103) $$\begin{array}{c} \alpha_{\lambda }=\gamma ,\;\;\beta_{\lambda }=\delta_{L}{\left(\iota^{\prime }\delta_{L}\right)}^{-1},\;\;\kappa_{\lambda }=\iota_{⊥}\delta_{R}\iota^{\prime }\delta_{L}\delta_{S}. \end{array}$$

Inserting the maximum likelihood estimators from Theorem 11 yields the estimators proposed by Lee and Carter. However, the disentangling of the singular values and singular vectors of $\hat{\delta }$ is done by the ad hoc identification *β*′*ι* = 1 and *κ*′*ι* = 0. These estimators are therefore specific to the considered data array and data set in parallel with the discussion in Sections 3.2 and 5.4.1.

#### 6.1.4. Consequences of the Possible Rank Deficiency

The parameter space *M* was split into spaces *M*_1_ and *M*_0_ depending on the rank of *δ*. The space *M*_0_ is a Lebesgue null set relative to *M*. Broadly speaking, there are two consequences of the possible rank deficiency. The first consequence is an estimation problem arising in the vicinity of *M*_0_. The second consequence is that the usual normal asymptotic distribution theory does not apply in the vicinity of *M*_1_. Whether this becomes a problem in practice depends on the data. One solution is to ensure that the time effect really is present when using the Lee-Carter model.

Investigate whether the time effects are present amounts to estimating the rank of *δ*. For a given data set two Lee-Carter models can be estimated. The first model with predictor space *M* is the unrestricted model in which rank⁡(*δ*) ≤ 1. The second model has predictor space *M*_0_ so *δ* = 0. Twice the difference of the likelihood values gives a likelihood ratio test statistic which is asymptotically *χ* ^2^. If the smaller model, *M*_0_, is accepted this is used in subsequent analysis. However, if the smaller model *M*_0_ is rejected then it is likely that the predictor is not located in the vicinity of *M*_0_ and it is then safe to work with the predictor space *M*_1_.

The consistency of this step-wise procedure is discussed in a cointegration context by Johansen [37, Section 12]. Even when this procedure points towards working with the parameter space *M*_1_ the rank deficiency may still affect inference under *M*_1_. Analysis of simple canonical correlation models suggests that inference under *M*_1_ will be nearly similar if the distance to *M*_0_ is sufficiently large. A problem is that the distribution for the test statistic will have poor finite sample properties when the parameter value is close to *M*_0_. A simple way to get around this problem is to test for *M*_0_ using a test with lower level than the conventional level. A more complicated way to address this is to employ a finite sample correction when seeking to test for *M*_0_. See Nielsen [38, 39] for further discussion of this issue in the context of simple canonical correlation models.

The rank deficiency issue is typically not encountered in a standard Lee-Carter analysis. The reason is that the analysis is typically applied to data where there is a marked improvement in mortality rates over time. A Lee-Carter analysis could however run into trouble if it were applied to data without a strong calendar effect. The issue becomes more pertinent when extending Lee-Carter model with a cohort component such as

(104) $$\begin{array}{c} \mu_{ij}=\beta_{i}^{\left(1\right)}+\beta_{i}^{\left(2\right)}\kappa_{j}^{\left(2\right)}+\beta_{i}^{\left(3\right)}\gamma_{j-i+I}^{\left(3\right)}; \end{array}$$

see Renshaw and Haberman [40]. If the cohort effect is modest the latter matrix is nearly rank deficient and the likelihood will be nearly flat in certain directions. This is presumably the reason for the estimation problem noted by Cairns et al. [41].

#### 6.1.5. Forecasting

The purpose of Lee-Carter model is usually to forecast future mortality. This issue is considered for the model with parameter space *M*_1_. The standard approach is to extrapolate *κ*, ad hoc identified through, for instance, *κ*′*ι* = 0. The *h*-step ahead extrapolation of *κ*_*J*+*h*_ based on some forecast methods is denoted by ${\tilde{\kappa }}_{J+h}(\hat{\kappa })$. Combined with the estimates ${\hat{\alpha }}_{i}$, ${\hat{\beta }}_{i}$ this gives the overall forecast

(105) $$\begin{array}{c} {\tilde{\mu }}_{i,J+h}\left(\hat{\theta }\right)={\hat{\alpha }}_{i}+{\hat{\beta }}_{i}{\tilde{\kappa }}_{J+h}\left(\hat{\kappa }\right). \end{array}$$

The identification question is then for which extrapolation methods this equals

(106) $$\begin{array}{c} {\tilde{\mu }}_{i,J+h}\left\{g_{1}\left(\hat{\theta }\right)\right\}=\left({\hat{\alpha }}_{i}+{\hat{\beta }}_{i}c\right)+\left(\frac{{\hat{\beta }}_{i}}{d}\right){\tilde{\kappa }}_{J+h}\left\{\left(\hat{\kappa }-c\right)d\right\}. \end{array}$$

The condition for avoiding adverse impact of the ad hoc identification is as follows.

**Theorem 12**

Let $\underline{\mu }\in M_{2}$. The forecast ${\tilde{\mu }}_{i,J+h}$ in (105) is invariant to ad hoc identification if and only if the extrapolation method for the period effect is location-scale preserving:

(107) $$\begin{array}{c} {\tilde{\kappa }}_{J+h}\left\{\left(\hat{\kappa }-c\iota \right)d\right\}=\left\{{\tilde{\kappa }}_{J+h}\left(\hat{\kappa }\right)-c\right\}d\;\forall c\in R,\;\forall d\neq 0. \end{array}$$

The default forecast method in the literature is a random walk with a drift, which was the preferred forecast of Lee and Carter [3]. This is given by

(108) $$\begin{array}{c} {\tilde{\kappa }}_{J+h}={\tilde{\kappa }}_{J+h-1}+\nu_{c}+\varepsilon_{h}, \end{array}$$

with estimates ${\hat{\nu }}_{c}={\left(J-1\right)}^{-1}\sum_{j=2}^{J}\left({\hat{\kappa }}_{j}-{\hat{\kappa }}_{j-1}\right)$ and normal errors *ε*_*h*_ with mean zero and estimated variance ${\hat{\sigma }}^{2}(\hat{\kappa })={\left(J-2\right)}^{-1}\sum_{j=2}^{J}{\left({\hat{\kappa }}_{j}-{\hat{\kappa }}_{j-1}-{\hat{\nu }}_{c}\right)}^{2}$. This extrapolation method is location-scale preserving as required in Theorem 12. It is even linear trend preserving. Other valid forecasts are a random walk without intercept as given by the equation ${\tilde{\kappa }}_{J+h}={\tilde{\kappa }}_{J+h-1}+\varepsilon_{h}$, or an autoregression given by ${\tilde{\kappa }}_{J+h}=\rho {\tilde{\kappa }}_{J+h-1}+\nu_{c}+\varepsilon_{h}$.

An alternative approach to forecasting would consider the predictor of the model for a particular age ground, say *i*. That predictor is ${\hat{\underline{\mu }}}_{i}=e_{i}^{\prime }(\hat{\gamma }\iota^{\prime }+\hat{\delta }\iota_{⊥}^{\prime })$, where *e*_*i*_ is the *i*th unit vector. From this we can generate forecasts ${\tilde{\mu }}_{i,J+h}$ using any time series method. The resulting forecast will in general depend on $\hat{\kappa }$ as well as ${\hat{\alpha }}_{i}$, ${\hat{\beta }}_{i}$ and it is therefore more general than the forecasts discussed in Theorem 12, which only depends on $\hat{\kappa }$. The forecast for another age group, say *i* ^†^, should be the same up to a linear transformation dictated by the Lee-Carter structure. Thus, the *h*-step ahead forecasts for the entire array are

(109) $$\begin{array}{c} {\tilde{\underline{\mu }}}_{J+h}=\frac{\hat{\delta }\iota_{⊥}^{\prime }e_{\overset{˘}{ı}}}{e_{i}^{\prime }\hat{\delta }\iota_{⊥}^{\prime }e_{\overset{˘}{ı}}}\left({\tilde{\mu }}_{i,J+h}-e_{i}^{\prime }\hat{\gamma }\iota^{\prime }\right)+\hat{\gamma }\iota^{\prime }, \end{array}$$

for an index $\overset{˘}{ı}$ is chosen so that $e_{i}^{\prime }\hat{\delta }\iota_{⊥}^{\prime }e_{\overset{˘}{ı}}\neq 0$.

#### 6.1.6. Bayesian Ad Hoc Identification Using a Dynamic Model

A Bayesian model with dynamic specification of the prior has been suggested by Pedroza [42]. Dynamic priors are presented for the time effects *θ* = (*ξ*, *λ*) involving a hyper parameter *ϕ*. The ad hoc identification (91) is imposed so that analysis is made for an ad hoc identified time effect *θ*_*λ*_.

Pedroza presents posteriors for *θ*_*λ*_. When evaluating this posterior one should bear in mind that the conditional prior *p*(*λ* | *ξ*) is not updated by the data; see Theorem 2. The presented extrapolative method does not depend on *λ*. Even so, the forecast will depend on conditional prior *p*(*ϕ* | *ξ*) which is not updated by the data; see Theorem 3.

### 6.2. The Two-Sample Lee-Carter Model

We now turn to applications of the Lee-Carter model in two-sample problems. Suppose two samples are for women and men. One approach would be to fit separate Lee-Carter models to the two datasets. These Lee-Carter models are of the form

(110) $$\begin{array}{c} \mu_{ijr}=\alpha_{ir}+\beta_{ir}\kappa_{jr}\;\text{for}\;\;i,j\in I_{\text{ap}},\;r=1,2. \end{array}$$

The objective is now to extrapolate the period effects *κ*_*jr*_. Extrapolating the two models separately using separate random walks is often seen to be volatile, so methods that seek to combine information from both estimated series ${\hat{\kappa }}_{jr}$ are sought after. The next result describes the invariance problem in forecasting.

**Theorem 13**

Let ${\underline{\mu }}_{r}\in M_{2r}$ for *r* = 1,2. The forecast ${\tilde{\mu }}_{i,J+h,1}$ for sample *r* = 1 is invariant to ad hoc identification if the extrapolation method ${\tilde{\kappa }}_{J+h,1}$ preserves location/scale for sample 1, but is invariant to location and scale for sample 2. That is for all *c*_1_, *c*_2_ ∈ *R* and all *d*_1_, *d*_2_ ≠ 0; then

(111) $$\begin{array}{c} {\tilde{\kappa }}_{J+h,1}\left\{d_{1}\left({\hat{\kappa }}_{1}-c_{1}\right),d_{2}\left({\hat{\kappa }}_{2}-c_{2}\right)\right\}=d_{1}\left\{{\tilde{\kappa }}_{J+h,1}\left({\hat{\kappa }}_{1},{\hat{\kappa }}_{2}\right)-c_{1}\right\}. \end{array}$$

For one sample the standard forecasting technique appears to be the random walk with a drift as in (108). For the two-sample problem a suggestion could be that women and men should share a common random walk with a drift but deviate from this by a stationary process. In econometrics this idea is referred to as cointegration as proposed by Engle and Granger [43]; see also Johansen [37] for a likelihood based vector autoregressive approach. It is tempting to require that the calendar effects should cointegrate with coefficients of unity, so *κ*_*j*1_ − *κ*_*j*2_ should be stationary. However, that apparently intuitive choice violates Theorem 13 because the locations and scales of *κ*_*jr*_ are different and arbitrary.

There are two fixes to this problem. The first solution is to work directly with the mortality predictors *μ*_*ijr*_ for an arbitrary age group *i* as outlined for the one-sample case in connection with (109). Since no identification is involved it is permitted to impose that *μ*_*ij*1_ and *μ*_*ij*2_ cointegrate with coefficients of unity. The forecast for age group *i* is then carried over to other age groups. The second solution is to work with the estimated series ${\hat{\kappa }}_{jr}$ but estimate the cointegrating coefficients from the data. In other words, the cointegrating relation ${\hat{\kappa }}_{j1}-\varphi {\hat{\kappa }}_{j2}-\psi$ should be zero mean, stationary, with coefficients *φ*, *ψ* estimated from the data. This can, for instance, be done by Johansen's approach for a bivariate vector autoregression; see Hendry and Nielsen [44, Section 17].

## 7. Conclusion

Ad hoc identification is intimately linked to interpretation, inference, numerical analysis, and forecasting. The ad hoc identification will often introduce an arbitrary element in the statistical analysis, whether it is based on frequentist or Bayesian methods. This arbitrary element is entirely avoidable and is in our view best avoided unless there is a clear substantial motivation for ad hoc identification. For decades there has been a debate over how it is best to ad hoc identify mortality models. Our proposal is to bypass this discussion by analysing the surjective mapping between the unidentified time effect parameter and the predictor of the model and then deduce a maximal invariant parametrisation. In our experience there are typically two substantial benefits. First, it simplifies estimation and other statistical computations which is what we have focused on here. Secondly and perhaps more importantly, it helps to focus the substantial question that gives rise to the analysis in the first place.

The issue of dealing with two time scales also occurs in other statistical models, such as the Cox regression model; see Cabrera et al. [45] for a recent application. In future research it would be interesting to consider whether the analysis presented here has any bearing on that problem.

## Appendix

### A. Proofs

#### A.1. Some Linear Algebra Results

Lemma A.1 Consider *A* ∈ *R* ^*q*×*p*^ and *C* ∈ *R* ^*q*×(*q*−*q*_*C*_)^ so *p*, *q*_*C*_ ≤ *q*. Suppose they have full column rank. Then the following statements are equivalent for some *p*_*C*_ ≤ *p*:

- (*A*, *C*) ∈ *R* ^*q*×(*p*+*q*−*q*_*C*_)^ has rank *p*_*C*_ + *q* − *q*_*C*_;
- *A*_⊥_′*C* ∈ *R* ^(*q*−*p*)×(*q*−*q*_*C*_)^ has rank *p*_*C*_ + *q* − *q*_*C*_ − *p*;
- *C*_⊥_′*A* ∈ *R* ^*q*_*C*_×*p*^ has rank *p*_*C*_.

Proof of Lemma A.1
(i)⇔(ii) Premultiply the matrix (*A*, *C*) with the invertible matrix ${\left(\bar{A},A_{⊥}\right)}^{\prime }$ to get the identity

(A.1) (A¯′A⊥′)(A,C)=(IpA¯′C0A⊥′C)=(Ip00A⊥′C)Mwhere  M=(IpA¯′C0Ip+q−qC).

Since the first matrix ${\left(\bar{A},A_{⊥}\right)}^{\prime }$ and the last matrix *M* have full rank then

(A.2) $$\begin{array}{c} \mathrm{rank}\left(A,C\right)=\mathrm{rank}\left(I_{p}\right)+\mathrm{rank}\left(A_{⊥}^{\prime }C\right)=p+\mathrm{rank}\left(A_{⊥}^{\prime }C\right). \end{array}$$

(i)*⇔*(iii) Swap the roles of *A*, *C* so rank⁡(*A*, *C*) = *q* − *q*_*C*_ + rank⁡(*C*_⊥_′*A*).

Lemma A.2 Consider *A* ∈ *R* ^*q*×*p*^ and *C* ∈ *R* ^*q*×(*q*−*q*_*C*_)^ so *p* ≤ *q*_*C*_ ≤ *q*. Suppose (*A*, *C*) ∈ *R* ^*q*×(*p*+*q*−*q*_*C*_)^ has full column rank *p* + *q* − *q*_*C*_. Then *m* = *A*_⊥_′*C* ∈ *R* ^(*q*−*p*)×(*q*−*q*_*C*_)^ has full column rank and (*A*, *C*) has orthogonal complement given by (*A*, *C*)_⊥_ = *A*_⊥_ *m*_⊥_ where *m*_⊥_ ∈ *R* ^(*q*−*p*)×(*q*_*C*_−*p*)^.

Proof of Lemma A.2
Since (*A*, *C*) has full column rank Lemma A.1 (i, ii) implies that *m* has full column rank. Since *m*_⊥_′*A*_⊥_′(*A*, *C*) = (*m*_⊥_′*A*_⊥_′*A*, *m*_⊥_′*m*) = 0 we argue that (*A*, *C*, *A*_⊥_ *m*_⊥_) is invertible. Premultiply by the invertible matrix ${\left(\bar{A},A_{⊥}\right)}^{\prime }$ to get

(A.3) $$\begin{array}{c} \left(\begin{array}{c} {\bar{A}}^{\prime }\\ A_{⊥}^{\prime } \end{array}\right)\left(A,C,A_{⊥}m_{⊥}\right)=\left(\begin{array}{ccc} I_{p} & {\bar{A}}^{\prime }C & 0\\ 0 & m & A_{⊥}^{\prime }A_{⊥}m_{⊥} \end{array}\right). \end{array}$$

The matrix (*m*, *A*_⊥_′*A*_⊥_ *m*_⊥_) has full rank. Indeed, its inverse is

(A.4) (m,A⊥′A⊥m⊥)−1=[(A⊥′A⊥)−1m{m′(A⊥′A⊥)−1m}−1,m⊥(m⊥′A⊥′A⊥m⊥)−1]′.

Thus, the block triangular matrix (A.3) and, hence, (*A*, *C*, *A*_⊥_ *m*_⊥_), have full rank.

#### A.2. Proofs of Main Theorems

Proof of Theorem 1
Since (*A*, *C*) has full column rank then Lemma A.2 shows it has orthogonal complement *A*_⊥_ *m*_⊥_ so that (*A*, *C*, *A*_⊥_ *m*_⊥_) is invertible. The orthogonal projection identity shows

(A.5) θ=(A,C¯)(A′C′)θ+A⊥m⊥(A⊥m⊥¯)′θ=(A,C¯)(ξψ)+A⊥m⊥ζ,

by the constraint *C*′*θ* = *ψ* and the definitions *A*′*θ* = *ξ* and ${\left(\bar{A_{⊥}m_{⊥}}\right)}^{\prime }\theta =\zeta$. This defines the constrained time effect space Θ_*C*_. Consider now the mapping *θ* ↦ *μ* = *XA*′*θ*. Premultiply the above expression for *θ* by *A*′ = (*I*_*p*_, 0)(*A*, *C*)′ to get *A*′*θ* = *ξ* so *μ* = *Xξ*. Thus, since *ψ* is fixed, the equivalence classes in Θ_*C*_ are given by *g*_*C*_ : *θ* ↦ *A*_⊥_ *m*_⊥_ *ζ*, with *ξ* = *A*′*θ* as a maximal invariant.

Proof of Theorem 2
(i) With the likelihood (32) so *p*(*y* | *ξ*, *λ*) = *p*(*y* | *ξ*) then

(A.6) p(y)=∫{∫p(y ∣ ξ,λ)p(ξ,λ)dλ}dξ=∫p(y ∣ ξ){∫p(ξ,λ)dλ}dξ=∫p(y ∣ ξ)p(ξ)dξ.

(ii) By Bayes formula and the likelihood (32) then

(A.7) $$\begin{array}{c} p\left(\xi \;|\;y\right)=\frac{p\left(y\;|\;\xi \right)p\left(\xi \right)}{p\left(y\right)},\\ p\left(\lambda \;|\;\xi ,y\right)=\frac{p\left(y\;|\;\xi ,\lambda \right)p\left(\lambda \;|\;\xi \right)}{p\left(y\;|\;\xi \right)}=p\left(\lambda \;|\;\xi \right). \end{array}$$

(iii) The posterior means are

(A.8) E(ξ ∣ y)=∫ξp(ξ ∣ y)dξ,E(λ ∣ ξ,y)=∫λp(λ ∣ ξ,y)dλ=∫λp(λ ∣ ξ)dλ,E(λ ∣ y)=∫λp(λ ∣ y)dλ=∫λ{∫p(λ,ξ ∣ y)dξ}dλ=∫p(ξ ∣ y){∫λp(λ ∣ ξ)dλ}dξ,

noting that *p*(*λ* | *ξ*, *y*) = *p*(*λ* | *ξ*).

Proof of Theorem 3
Consider the expressions in (37) and (38); that is,

(A.9) $$\begin{array}{c} p\left(\tilde{\mu }\;|\;y\right)=\iint p\left(\tilde{\mu }\;|\;\xi ,\lambda ,y\right)p\left(\xi \;|\;y\right)p\left(\lambda \;|\;\xi \right)d\lambda \;d\xi \end{array}$$

and a similar expression involving *p* ^†^. The question is when they are identical. Assuming $p(\tilde{\mu }|\xi ,\lambda ,y)=p(\tilde{\mu }|\xi ,y)$ as in (39) the expression reduces to

(A.10) p(μ~ ∣ y)=∫p(μ~ ∣ ξ,y)p(ξ ∣ y){∫p(λ ∣ ξ)dλ}dξ=∫p(μ~ ∣ ξ,y)p(ξ ∣ y)dξ,

since the conditional prior integrates to unity. The same applies for the expression involving *p* ^†^(*λ* | *ξ*).  

Proof of Theorem 5
Similar to the proof of Kuang et al. [19, Theorem 1], albeit for a rectangular instead of a triangular data array.

Proof of Theorem 8
Recall *θ*_intrisic_ = *C*_*S*⊥_ *C*_*S*⊥_′*A*(*A*′*C*_*S*⊥_ *C*_*S*⊥_′*A*)^−1^ *ξ* where *C*_*S*⊥_ = *C*_⊥_(*S*_⊥_′*C*_⊥_)^−1^ as defined in (72). Premultiply by *A*′ and *C*′ to see that *A*′*θ*_intrisic_ = *ξ* and *C*′*θ*_intrisic_. This does, however, not describe the full variation of *θ*_intrisic_ since (*A*, *C*) is not a square matrix. We must extend the matrix (*A*, *C*) with columns so that it is square and invertible. We find a vector *w* ∈ *R* ^*q*−3^ so that (*A*, *C*, *C*_⊥_ *w*) is invertible. Recall *A* ∈ *R* ^*q*×*p*^ where *q* = *p* + 4. The matrix *C* ∈ *R* ^*q*×3^ is chosen so that (*A*, *C*) has full column rank *q* − 1 = *p* + 3 as discussed in Section 5.4.1. Apply Lemma A.2, swapping the role of *A*, *C*, to see *w*_⊥_ = *C*_⊥_′*A*, say, has full column rank. Then (*A*, *C*) has orthogonal complement *C*_⊥_ *w*. We show that for any invertible matrix *M* ∈ *R* ^*q*×*q*^ then the 1 × 2 block matrix {(*A*, *C*), *MC*_⊥_ *w*} is invertible. To see this hold, premultiply by {(*A*, *C*), *C*_⊥_ *w*}′ to see that an invertible upper triangular matrix arises. To analyse the properties of *θ*_intrisic_ it suffices to analyse {(*A*, *C*), *MC*_⊥_ *w*}′*θ*_intrisic_, since there is a bijective mapping between the two. Choose $M={\bar{C}}_{⊥}(C_{⊥}^{\prime }S_{⊥})(S_{⊥}^{\prime }C_{⊥}){\bar{C}}_{⊥}^{\prime }+CC^{\prime }$. Then it holds that $C_{⊥}^{\prime }M^{\prime }=(C_{⊥}^{\prime }S_{⊥})(S_{⊥}^{\prime }C_{⊥}){\bar{C}}_{⊥}^{\prime }$ so that *C*_⊥_′*M*′*C*_*S*⊥_ *C*_*S*⊥_′ = *C*_⊥_′ and therefore

(A.11) $$\begin{array}{c} w^{\prime }C_{⊥}^{\prime }M^{\prime }\theta_{\text{intrisic}}=w^{\prime }C_{⊥}^{\prime }A{\left(A^{\prime }C_{S⊥}C_{S⊥}^{\prime }A\right)}^{-1}\xi =0, \end{array}$$

since *w*′*C*_⊥_′*A* = 0 by construction. Thus, it holds that $w^{\prime }(C_{⊥}^{\prime }S_{⊥})(S_{⊥}^{\prime }C_{⊥}){\bar{C}}_{⊥}^{\prime }\theta_{\text{intrisic}}=0$ as required.

Proof of Theorem 9
This is a generalisation of the proof of Kuang et al. [25, Theorems 1,2]. Let *k*_*h*_ = *J* + *h* + *I* − *i* − *K* = *h* − *i* − 1. (i) Recall the group *g* in (57). Then (i) follows by comparing the equations

(A.12) μ~i,J+h(θ^)=α^i+β~J+h(θ^)+γ~K+kh(θ^)+δ^,μ~i,J+h{g(θ^)}=α^i+a+di+β~J+h{g(θ^)}+γ~K+kh{g(θ^)} +δ^−a−b−c−dI.

(ii) As in Section 3.1 there is a bijective mapping from *θ* to *λ*, *ξ*, where *ξ* is invariant to *g*, but *λ* is not. The choice of *λ* is not important. Any extrapolations of ${\hat{\beta }}_{j}$, ${\hat{\gamma }}_{k}$ can then be written in the form

(A.13) $$\begin{array}{c} {\tilde{\beta }}_{J+h}\left(\hat{\theta }\right)={\hat{\beta }}_{J}+h\Delta {\hat{\beta }}_{J}+F_{\beta }\left(\hat{\lambda },\hat{\xi }\right),\\ {\tilde{\gamma }}_{K+k_{h}}\left(\hat{\theta }\right)={\hat{\gamma }}_{K}+k_{h}\Delta {\hat{\gamma }}_{K}+F_{\gamma }\left(\hat{\lambda },\hat{\xi }\right), \end{array}$$

for some functions *F*_*β*_, *F*_*γ*_. Applying the group *g* it follows

(A.14) $$\begin{array}{c} {\tilde{\beta }}_{J+h}\left\{g\left(\hat{\theta }\right)\right\}={\hat{\beta }}_{J}+b-dJ+h\left(\Delta {\hat{\beta }}_{J}-d\right)+F_{\beta }\left\{g\left(\hat{\lambda }\right),\hat{\xi }\right\},\\ {\tilde{\gamma }}_{K+k_{h}}\left\{g\left(\hat{\theta }\right)\right\}={\hat{\gamma }}_{K}+c+dK+k_{h}\left(\Delta {\hat{\gamma }}_{K}+d\right)+F_{\gamma }\left\{g\left(\hat{\lambda }\right),\hat{\xi }\right\}. \end{array}$$

Due to (i) it must hold *F*(*λ*, *ξ*) = *F*_*β*_(*λ*, *ξ*) + *F*_*γ*_(*λ*, *ξ*) and must equal *F*{*g*(*λ*), *ξ*} = *F*_*β*_{*g*(*λ*), *ξ*} + *F*_*γ*_{*g*(*λ*), *ξ*}. The function *F* must then be constant in the first argument. This must apply in the forecast region *J*_ap_ where *γ* is not extrapolated. Therefore, it must also hold that the *F*_*β*_(*λ*, *ξ*) = *F*_*β*_{*g*(*λ*), *ξ*}, and in turn that *F*_*γ*_(*λ*, *ξ*) = *F*_*γ*_{*g*(*λ*), *ξ*}. Conversely, if the functions *F*_*β*_, *F*_*γ*_ are constant in *λ* then the forecast is invariant to *g*.

Proof of Theorem 10
(i) Equation (95) shows that *ξ* is a function of *θ*. (ii) Equation (93) shows that $\underline{\mu }=\gamma \iota^{\prime }+\delta \iota_{⊥}^{\prime }$ is a function of *ξ*. (iii) The decomposition $\underline{\mu }=\underline{\mu }\bar{\iota }\iota^{\prime }+\underline{\mu }{\bar{\iota }}_{⊥}^{\prime }\iota_{⊥}$ shows that there is a one-one mapping between $\underline{\mu }$ and $\left(\underline{\mu }\bar{\iota },\underline{\mu }{\bar{\iota }}_{⊥}^{\prime }\right)$. In turn, (93) shows that $\left(\underline{\mu }\bar{\iota },\underline{\mu }{\bar{\iota }}_{⊥}^{\prime })=(\gamma ,\delta \right)$. Thus if (*γ* ^†^, *δ* ^†^)≠(*γ* ^‡^, *δ* ^‡^) then ${\underline{\mu }}^{†}\neq {\underline{\mu }}^{‡}$.

Proof of Theorem 11
Rewrite the trace term using the identity $I_{J}=\bar{\iota }\iota^{\prime }+{\bar{\iota }}_{⊥}\iota_{⊥}^{\prime }$ to get

(A.15) T=tr⁡{(Y_−μ_)(Y_−μ_)′}=tr⁡{(Y_−μ_)ι¯ι′(Y_−μ_)′} +tr⁡{(Y_−μ_)ι¯⊥ι⊥′(Y_−μ_)′}.

By (94) then $\underline{\mu }=\gamma \iota^{\prime }+\delta \iota_{⊥}^{\prime }$ so that *T* = *T*_1_ + *T*_2_ where

(A.16) T1=tr⁡{(Y_−γι′)ι¯ι′(Y_−γι′)′}=ι′ιtr⁡{(Y_ι¯−γ)(Y_ι¯−γ)′},T2tr⁡{(Y_−δι⊥′)ι¯⊥ι⊥′(Y_−δι⊥′)′}=tr⁡{(Y_ι¯⊥ι⊥′−δι⊥′)(Y_ι¯⊥ι⊥′−δι⊥′)′}.

The term *T*_1_ has a minimum of zero if and only if $\underline{Y}\bar{\iota }=\gamma$. In *T*_2_ replace for a moment *δι*_⊥_′ by a matrix *ϕ* with rank of at most one. The altered *T*_2_ is minimised when *ϕ* is the singular value decomposition of $\underline{Y}{\bar{\iota }}_{⊥}\iota_{⊥}^{\prime }$ truncated to rank one due; see Golub and van Loan [46, Theorem 2.5.2]. That singular value decomposition has the property that it is zero when multiplied by *ι*. Therefore it is also the minimiser of the original problem.

Proof of Theorem 12

It follows by comparing (105) and (106).

Proof of Theorem 13
Write ${\tilde{\mu }}_{i,J+h,1}({\hat{\theta }}_{1},{\hat{\theta }}_{2})={\hat{\alpha }}_{i1}+{\hat{\beta }}_{i1}{\tilde{\kappa }}_{J+h,1}({\hat{\kappa }}_{1},{\hat{\kappa }}_{2})$. This is equals to ${\tilde{\mu }}_{i,J+h,1}\{g_{2}({\hat{\theta }}_{1}),g_{2}({\hat{\theta }}_{2})\}=({\hat{\alpha }}_{i1}+{\hat{\beta }}_{i1}c_{1})+({\hat{\beta }}_{i1}/d_{1}){\tilde{\kappa }}_{J+h,1}\{d_{1}({\hat{\kappa }}_{1}-c_{1}),d_{2}({\hat{\kappa }}_{2}-c_{2})\}$ under the given condition.

#### A.3. A Further Result on Time Effect Hypotheses

Consider the restriction *R*′*θ*_*R*_ = *ρ* of (28). The following result holds.

Theorem A.3 Consider the restriction

(A.17) $$\begin{array}{c} R^{\prime }\theta_{R}=\rho \end{array}$$

of (28) where *A* ∈ *R* ^*q*×*p*^ and *R* ∈ *R* ^*q*×(*q*−*q*_*R*_)^ so *p*, *q*_*R*_ ≤ *q*. Suppose *A* and *R* have full column rank. Then, for some *p*_*R*_ ≤ min⁡(*p*, *q*_*R*_) it holds *p*_*R*_ = rank⁡(*R*_⊥_′*A*) = rank⁡(*A*, *R*)−(*q* − *q*_*R*_). Then write *R*_⊥_′*A* = *ab*′ for some matrices *a* ∈ *R* ^*q*_*R*_×*p*_*R*_^ and *b* ∈ *R* ^*p*×*p*_*R*_^ with full column rank. The hypothesis (28) restricts the canonical parameter affinely through

(A.18) $$\begin{array}{c} b_{⊥}^{\prime }\xi =b_{⊥}^{\prime }A^{\prime }\bar{R}\rho , \end{array}$$

so that the degrees of freedom of the restriction is *p* − *p*_*R*_. Introduce the parameters

(A.19) $$\begin{array}{c} \varphi_{1}=a^{\prime }{\bar{R}}_{⊥}^{\prime }\theta_{R},\;\;\varphi_{2}={\bar{b}}^{\prime }A^{\prime }\xi =\varphi_{1}+{\bar{b}}^{\prime }A^{\prime }\bar{R}\rho . \end{array}$$

Then, the predictor *μ* can be written as the *p*_*R*_-dimensional affine subspace of the form

(A.20) MR=(μ∈Rn:μ=Xbφ1+XA′R¯ρ  for  φ1∈RpR)

(A.21) =(μ∈Rn:μ=Xbφ2+Xb¯⊥b⊥′A′R¯ρ  for  φ2∈RpR).

The mapping *θ*_*R*_ ↦ *μ* = *XA*′*θ*_*R*_ on *θ*_*R*_ ∈ Θ_*R*_ is invariant with respect to the group *g*_*R*_ : *θ*_*R*_ ↦ *θ*_*R*_ + *R*_⊥_ *a*_⊥_ *ζ*_3_ with *φ*_1_ and *φ*_2_ as maximal invariants. A special case arises if the restriction combines a restriction on *A* with ad hoc identification. That is, if *R* = (*Ad*, *C*) where *d* ∈ *R* ^*p*×(*p*−*p*_*R*_)^ and *C* ∈ *R* ^*q*×{(*q*−*p*)−(*q*_*R*_−*p*_*R*_)}^ so (*A*, *C*) has full column rank. Then *b* = *d*_⊥_ so that the restriction (A.18) reduces to *d*′*ξ* = (*I*_*p*−*p*_*R*__, 0)*ρ*.

Proof of Theorem A.3
Apply Lemma A.1 (i, iii) to see that rank⁡(*A*, *R*) = *p*_*R*_ + *q* − *q*_*R*_ is equivalent to rank⁡(*R*_⊥_′*A*) = *p*_*R*_. We can then write *R*_⊥_′*A* = *ab*′ for some matrices *a* ∈ *R* ^*q*_*R*_×*p*_*R*_^ and *b* ∈ *R* ^*p*×*p*_*R*_^ with full column rank. Exploit the projection identity $I_{q}=R_{⊥}{\bar{R}}_{⊥}^{\prime }+\bar{R}R^{\prime }$ to get $\xi =A^{\prime }\theta_{R}=A^{\prime }R_{⊥}{\bar{R}}_{⊥}^{\prime }\theta_{R}+A^{\prime }\bar{R}R^{\prime }\theta_{R}$. Insert *A*′*R*_⊥_ = *ba*′ and *R*′*θ*_*R*_ = *ρ* to get $\xi =ba^{\prime }{\bar{R}}_{⊥}^{\prime }\theta_{R}+A^{\prime }\bar{R}\rho$ and therefore

(A.22) $$\begin{array}{c} \theta ⟼\mu =XA^{\prime }\theta =Xba^{\prime }{\bar{R}}_{⊥}^{\prime }\theta_{R}+XA^{\prime }\bar{R}\rho . \end{array}$$

Inserting the orthogonal projection $I_{p}=b{\bar{b}}^{\prime }+{\bar{b}}_{⊥}b_{⊥}^{\prime }$ this can also be written as

(A.23) $$\begin{array}{c} \theta ⟼\mu =XA^{\prime }\theta =Xb\left(a^{\prime }{\bar{R}}_{⊥}^{\prime }\theta_{R}+{\bar{b}}^{\prime }A^{\prime }\bar{R}\rho \right)+X{\bar{b}}_{⊥}b_{⊥}^{\prime }A^{\prime }\bar{R}\rho . \end{array}$$

Noting that $\varphi_{1}=a^{\prime }{\bar{R}}_{⊥}^{\prime }\theta_{R}$ is freely varying so is $\varphi_{2}=a^{\prime }{\bar{R}}_{⊥}^{\prime }\theta_{R}+{\bar{b}}^{\prime }A^{\prime }\bar{R}\rho$. To rewrite *φ*_2_ premultiply the first term by $I_{p_{R}}={\bar{b}}^{\prime }b$ to get $\varphi_{2}={\bar{b}}^{\prime }(ba^{\prime }{\bar{R}}_{⊥}^{\prime }\theta_{R}+A^{\prime }\bar{R}\rho )$. Then insert *ρ* = *R*′*θ*_*R*_ and *ba*′ = *A*′*R*_⊥_ to get $\varphi_{2}={\bar{b}}^{\prime }A^{\prime }(R{\bar{R}}_{⊥}^{\prime }+\bar{R}R^{\prime })\theta_{R}={\bar{b}}^{\prime }A^{\prime }\theta_{R}={\bar{b}}^{\prime }A^{\prime }\xi$. This gives the space *M*_*R*_ in (A.21). To derive the reduced group *g*_*R*_ choose a *θ*_*R*_ ∈ Θ_*R*_ so that *R*′*θ*_*R*_ = *ρ*. Any *θ*_*R*_^†^ ∈ Θ can be written as $\theta_{R}^{†}=\theta_{R}+\bar{R}\zeta_{1}+R_{⊥}\bar{a}\zeta_{2}+R_{⊥}a_{⊥}\zeta_{3}$ since $\left(\bar{R},R_{⊥}\right)$ and $\left(\bar{a},a_{⊥}\right)$ have full rank. Since *θ*_*R*_ ∈ Θ_*R*_ then *θ*_*R*_^†^ ∈ Θ_*R*_ if and only if *ζ*_1_ = 0. We now consider whether *θ*_*R*_, *θ*_*R*_^†^ are equivalent with respect to the restricted mapping (A.22). It holds

(A.24) $$\begin{array}{c} \theta_{R}^{†}⟼\mu^{†}=Xba^{\prime }{\bar{R}}_{⊥}^{\prime }\left(\theta_{R}+R_{⊥}\bar{a}\zeta_{2}+R_{⊥}a_{⊥}\zeta_{3}\right)+XA^{\prime }\bar{R}\rho . \end{array}$$

Noting that $\mu =Xba^{\prime }{\bar{R}}_{⊥}^{\prime }\theta_{R}+XA^{\prime }\bar{R}\rho$ and $a^{\prime }{\bar{R}}_{⊥}^{\prime }R_{⊥}\bar{a}=I_{p_{R}}$, while $a^{\prime }{\bar{R}}_{⊥}^{\prime }R_{⊥}a_{⊥}=0$, then *μ* ^†^ = *μ* + *Xbζ*_2_ which reduces to *μ* if and only if *ζ*_2_ = 0. Thus, the mapping *θ*_*R*_ ↦ *μ* on *θ*_*R*_ ∈ Θ_*R*_ is invariant with respect to the group *g*_*R*_ : *θ*_*R*_ ↦ *θ*_*R*_ + *R*_⊥_ *a*_⊥_ *ζ*_3_ with *φ*_1_ and *φ*_2_ as maximal invariants. Now, suppose *R* = (*Ad*, *C*). Then Lemma A.2 shows that *m* = *A*_⊥_′*C* has full column rank, while (*A*, *C*) has orthogonal complement *A*_⊥_ *m*_⊥_. Thus, *R* has orthogonal complement

(A.25) R⊥={(A,C¯)(d⊥0),(A,C)⊥}={(A,C¯)(Ip0)d⊥,A⊥m⊥}.

In particular, it holds *A*′*R*_⊥_ = (*I*_*p*_, 0)(*A*, *C*)′*R*_⊥_ = (*d*_⊥_, 0) = *d*_⊥_(*I*_*p*_*R*__, 0), which is denoted *ba*′ in the general case, so that *b* = *d*_⊥_. Consider the restriction (A.18). Here, *b*_⊥_′*ξ* = *d*′*ξ*, while *b*_⊥_′*A*′ = *d*′*A*′ = (*I*_*p*−*p*_*R*__, 0)(*Ad*, *C*)′ = (*I*_*p*−*p*_*R*__, 0)*R*′ so that $b_{⊥}^{\prime }A^{\prime }\bar{R}\rho =(I_{p-p_{R}},0)\rho$.
